# Comprehensive Transcriptomic Analysis of Novel Class I HDAC Proteolysis Targeting Chimeras (PROTACs)

**DOI:** 10.1021/acs.biochem.2c00288

**Published:** 2022-08-10

**Authors:** India M. Baker, Joshua P. Smalley, Khadija A. Sabat, James T. Hodgkinson, Shaun M. Cowley

**Affiliations:** † Department of Molecular and Cell Biology, University of Leicester, Leicester LE1 7RH, U.K.; ‡ Leicester Institute of Structural and Chemical Biology, School of Chemistry, University of Leicester, Leicester LE1 7RH, U.K.

## Abstract

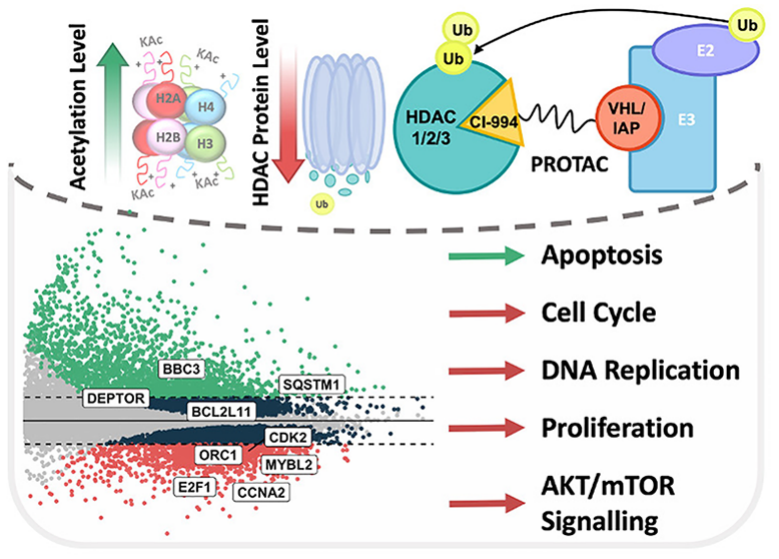

The class I histone deacetylase (HDAC) enzymes;HDAC1,2 and 3 form the catalytic engine of at least seven structurally distinct multiprotein complexes in cells. These molecular machines play a vital role in the regulation of chromatin accessibility and gene activity via the removal of acetyl moieties from lysine residues within histone tails. Their inhibition via small molecule inhibitors has beneficial effects in a number of disease types, including the clinical treatment of hematological cancers. We have previously reported a library of proteolysis targeting chimeras (PROTACs) incorporating a benzamide-based HDAC ligand (from CI-994), with an alkyl linker and ligand for the von Hippel-Lindau (VHL) E3 ubiquitin ligase that degrade HDAC1–3 at submicromolar concentrations. Here we report the addition of two novel PROTACs (JPS026 and JPS027), which utilize a ligand for the cellular inhibitor of apoptosis (IAP) family of E3 ligases. We found that both VHL (JPS004)- and IAP (JPS026)-based PROTACs degrade HDAC1–3 and induce histone acetylation to a similar degree. However, JPS026 is significantly more potent at inducing cell death in HCT116 cells than is JPS004. RNA sequencing analysis of PROTAC-treated HCT116 cells showed a distinct gene expression signature in which cell cycle and DNA replication machinery are repressed. Components of the mTORC1 and -2 complexes were also reduced, leading to an increase in FOXO3 and downstream target genes that regulate autophagy and apoptosis. In summary, a novel combination of HDAC and IAP ligands generates a PROTAC with a potent ability to stimulate apoptosis and differential gene expression in human cancer cells.

Nε-Lysine acetylation (Kac) of proteins is a common post-translational modification in all cells from bacteria and plants to humans.^1,2^ The addition of the acetyl moiety can occur as the result of a direct chemical reaction, particularly in mitochondria and chloroplasts (the engine rooms of the cell, where the cofactor acetyl-CoA is abundant) or via the action of lysine acetyltransferase enzymes, such as p300.^3^ Kac fundamentally changes the chemistry of the lysine, neutralizing its positive charge and extending the length of the side chain such that it becomes recognizable by proteins with a bromodomain.^4^ Critical to its role in modifying protein–protein interactions in cells, Kac is reversible. The acetyl group can be removed by two main families of deacetylase enzymes, the classical Zn^2+^-dependent histone deacetylases (HDAC1–11) and sirtuins (SIRT1–7). Thousands of proteins are acetylated in human cells,^5,6^ although intriguingly, most of these proteins are acetylated at a stoichiometry of <0.1% on average, the exception being histones.^7^ Histone tails are rich in Lys residues, and their acetylation relaxes the grip of the nucleosome on the local DNA, making it more accessible to transcription factors and the transcription machinery.^1^ HDACs thus play a key role in regulating chromatin accessibility across the genome. Indeed, given that the majority of Kac occurs in histones, this might be its main regulatory function in cells.

The class I HDAC enzymes (HDAC1–3) are found in the nucleus of all cell types as the catalytic heart of multiprotein complexes that regulate global histone Kac levels.^8^ HDAC3 forms a 1:1 complex with the nuclear receptor co-repressors NCoR1 and NCoR2 (SMRT).^9^ The highly related HDAC1 and HDAC2 (HDAC1/2), on the other hand, form the structural and catalytic components of at least seven different complexes,^8^ each with a unique function in cells. These HDAC1/2-based chromatin-modifying machines vary in their complexity, from the monomeric co-repressor of the REST (CoREST) complex^10^, to dimeric complexes such as the nucleosome remodeling and deacetylase (NuRD)^11^ and Swi-independent 3 (Sin3)^12^, to the tetrameric mitotic deacetylate complex (MiDAC).^13^ They are usually referred to as co-repressor complexes, although a number play roles in both active and repressed genes, while the main job of MiDAC might be cell cycle progression. Unsurprisingly, given the sheer number of different HDAC1/2 complexes, deletion of *Hdac1/2* is lethal in a range of cell types.^14^ Loss of HDAC1/2 in embryonic stem cells and T cells results in a 50% reduction of total HDAC activity,^15,16^ making them, biochemically at least, the dominant deacetylase enzymes in the cell. The essential nature of HDAC1–3 activity has led to the development of class I specific HDAC inhibitors (HDACi). Uniquely among the Zn-dependent HDACs, HDAC1–3 have an additional 14 Å of space adjacent to the catalytic site, large enough to accommodate the bulky aromatic group of benzamide-based inhibitors, such as CI-994 (tacedinaline) and MS-275 (entinostat). Treatment of cancer cells with CI-994 causes a deceleration of the cell cycle and an induction of apoptosis.^17,18^

In addition to classical HDACi that bind through the active site and chelate the Zn^2+^, we and others have begun to develop proteolysis targeting chimeras (PROTACs) to class I HDACs that both bind and degrade HDAC1/2 and -3 within the context of their respective complexes.^19,20^ PROTACs we developed incorporate a CI-994 molecule coupled to either von Hippel-Lindau (VHL) or cereblon E3 ligands, via a flexible linker. Surprisingly, we found that longer linkers (≥12 atoms) were cell permeable and more effective HDAC1/2 and 3 degraders than PROTACs with shorter linkers (at most nine atoms).^21^ Colon cancer cells were more sensitive to PROTACs JPS014 and JPS016 than to the parental molecule, CI-994.^22^ In this study, we define a brand-new class of HDAC1–3 degraders in which we have coupled CI-994 via a 12-carbon linker to a ligand for the inhibitor of apoptosis (IAP) family of E3 ligases, termed JPS026. Surprisingly, we discovered that HCT116 cells treated with JPS026 were significantly more sensitized to apoptosis at much lower concentrations than either the parental molecule, CI-994, or VHL-derived PROTACs (e.g., JPS016), while the IAP ligand alone had no effect. We observed robust degradation for both VHL- and IAP-derived PROTACs, although interestingly, JPS026 was a less potent degrader than JPS016, suggesting that an increased level of apoptosis might be caused by the unique combination of IAP and HDAC inhibition. Despite their clinical use, uncertainty about the mode of action of HDACi on cancer cells remains. To address this question in a colon cancer model, we have compared the transcriptomes of cells treated with CI-994 and seven different PROTACs. We find that the more potent HDAC modulators (i.e., those best able to promote histone hyperacetylation) correlate with the number of differentially expressed genes (DEGs). The number of DEGs produced decreases in the following order: JPS16 (VHL ligand) > CI994 > JPS026 (IAP ligand). This suggests that the increased level of apoptosis observed with JPS026 is not due solely to changes in gene expression. We have developed and characterized a novel IAP recruiting class of HDAC1–3 PROTACs and performed a comprehensive parallel transcriptomic analysis of PROTACs, providing a high-powered glimpse of the transcriptional events that occur upon HDAC inhibition in cancer cells.

## Materials and Methods

### Cell Culture and PROTAC Treatment

HCT116 cells obtained from ATCC (CCL-247) were maintained in Dulbecco’s modified Eagle’s medium (DMEM, Gibco, catalog no. 41963039), supplemented with 50 μL/mL [10% (v/v)] fetal bovine serum (FBS, Sigma, F9665) and 1.1 units/mL [1.1% (v/v)] penicillin-streptomycin-glutamine (Gibco, 10378016), and incubated at 37 °C and 5% CO_2_. For PROTAC treatments, HCT116 cells were seeded at a density of 4 × 10^5^ cells per well on six-well plates 24 h before treatment with class I HDAC targeting PROTACs. Cells were exposed to either VHL ligand-based PROTACs (JPS004, JPS014, JPS016, JPS039, and JPS036 dosed at 10 μM) or IAP ligand-based PROTACs (JPS026 and JPS027 dosed at 5 μM) as well as relevant controls (0.01% DMSO, 10 μM CI994, and 5 μM IAP ligand) for 24 h at the indicated concentrations.

### Synthesis and Characterization of PROTACs

The PROTAC library used during this study was synthesized in house as described previously by Smalley et al.^22^ The full experimental methods and characterization for the new IAP PROTACs JPS026 and JPS027 can be found in the Supporting Information.

### Western Blotting

Quantitative Western blotting was performed as outlined previously by Smalley et al.^22^ In brief, cell pellets harvested from PROTAC-treated HCT116 cells were snap-frozen (−196 °C) before being thawed and lysed with NP-40 lysis buffer (50 mM Tris-HCl, 150 mM NaCl, 0.5% NP-40, and 0.5% Triton X-100] supplemented with 1% (v/v) protease inhibitor cocktail (Sigma, P8340) for 30 min at 4 °C. Cell debris and DNA were then pelleted (14000 rpm, 15 min, 4 °C), and the protein lysate was collected. Histones were then extracted from the pelleted DNA via acid extraction with the addition of 0.4 N H_2_SO_4_ before overnight incubation at 4 °C. The protein concentration was determined using the Bio-Rad protein assay dye reagent (Bio-Rad, 5000006), and extracts were then denatured via the addition of a 4× loading dye [4× LDS loading buffer (NuPAGE, NP0007) and 4% (v/v) β-mercaptoethanol] and incubation at 95 °C for 5 min. For protein resolution, 30 μg of the protein lysate or 2.5 μg of the purified histone extract was resolved on 4% to 12% Bis-Tris sodium dodecyl sulfate–polyacrylamide gel electrophoresis gels (SDS;Invitrogen, NP0322) alongside a 10–250 kDa PageRuler Plus, prestained protein ladder (Thermo Scientific, 26619). Protein resolution and transfer onto nitrocellulose membranes were carried out using the XCell SureLock Mini-Cell and XCell II Blot Module (Invitrogen, EI0002) as per the manufacturer’s guidelines. Membranes were then probed with primary and secondary IRDye-conjugated antibodies (detailed in Table S1) before visualization using the LICOR Odessy imaging system. Image processing and quantification were carried out using the Image Studio Lite software.

### Cell Cycle and Viability Flow Cytometry

The cell cycle distribution of PROTAC-treated HCT116 cells was assessed using propidium iodide (PI) staining coupled with flow cytometry. Samples containing ∼100000 cells were fixed in 70% ethanol and stored at −20 °C for 24 h prior to staining. After being fixed, samples were washed twice in PBS and pelleted via centrifugation at 1100 rpm for 5 min to remove residual ethanol before being stained. Cells were then resuspended in 500 μL of a PI staining solution containing 50 μg/mL propidium iodide (Invitrogen, P3566) and 50 μg/mL DNase and protease-free RNase A (Thermo Scientific, EN0531), diluted in sterile PBS (Sigma, D8537), and incubated in the dark at 4 °C for 24 h. Flow cytometric analysis was then carried out on a BD Canto II flow cytometer equipped with a 488 nm laser line, resulting data were analyzed using FlowJo version 10.7. An example of the gating strategy employed is depicted in Figure S3.

### RNA Sequencing

RNA sequencing analysis was carried out to determine the differential gene expression profiles of HCT116 cells treated with VHL (JPS004, JPS014, JPS016, JPS036, and JPS039 dosed at 10 μM) and IAP (JPS026 and JPS027 dosed at 5 μM) E3 ubiquitin ligase-based class I HDAC targeting PROTACs. The total RNA was isolated from PROTAC-treated samples using a Trireagent-based RNA miniprep kit (Zymogen, R2053) following the manufacturer’s instructions. Isolated RNA was then subjected to quality control and quantification using an Agilent Bioanalyzer. mRNA library preparation (poly A enrichment) and sequencing were performed by Novogene (Cambridge, United Kingdom). mRNA sequencing was carried out at a read depth of 20 million, using the NovaSeq 6000 PE150 platform.

### Bioinformatics Analysis

Data were downloaded from Novogene and MD5Sums checked. Quality control steps were performed on raw and aligned data using FASTQC (version 0.11.9).^23^ Paired-end reads were mapped to the HISAT2 GRCh38_tran index build using HISAT2 (version 2.2.1),^24^ with default parameters. SAM files containing the aligned reads were then sorted and converted into binary format (BAM) before being indexed with SAMtools (version 1.12).^25^ To obtain the read counts across exons for each sample, the command-line program LiBiNorm (version 2.4)^26^ supplied with the “Homosapiens.GRCh38.104” GTF annotation file obtained from Ensembl was used. LiBiNorm was run in HT-Seq compatible using the following parameters: LiBiNorm count -z -r pos -i gene_name -s reverse <BAM file> Homo_sapiens.GRCh38.104.gtf <OUTPUT file>. Differential gene analysis and default modeling of expression values were carried out using the R package DESeq2 (version 1.31.16).^27^ Results were obtained using the DESeq2 lfcshrink() function employing the “apeglm” method for the visualization and ranking of genes for downstream analysis.^28^ Differentially expressed genes (DEGs) were then defined by significance thresholds of a *p*-adjusted value of <0.01 and a fold change of >2 (log_2_ fold change > 1) for downstream analysis. For gene ontology (GO) analysis of enriched biological processes, the Bioconductor package topGO (version 2.44.0.) and the human genome-wide annotation package org.Hs.eg.db (version 3.8.2) were used.^29,30^ The raw data and processed count files from this study can be obtained from the Gene Expression Omnibus (GEO) database (https://www.ncbi.nlm.nih.gov/geo/) under accession number GSE197985.

### Statistical Analysis

All quantitative Western blot and flow cytometry values are presented as the mean ± standard deviation (SD) of three independent biological replicates. Quantitative Western blot data from the histone marks H3K56AC and H2BK5Ac were analyzed using one-way analysis of variance (ANOVA) with Dunnett’s correction. A *p* value of ≤0.05 was deemed significant.

## Results

### Generation of Novel Small Molecule Protein Targeting Chimeras (PROTACs) for Class I HDACs

**Figure 1:**
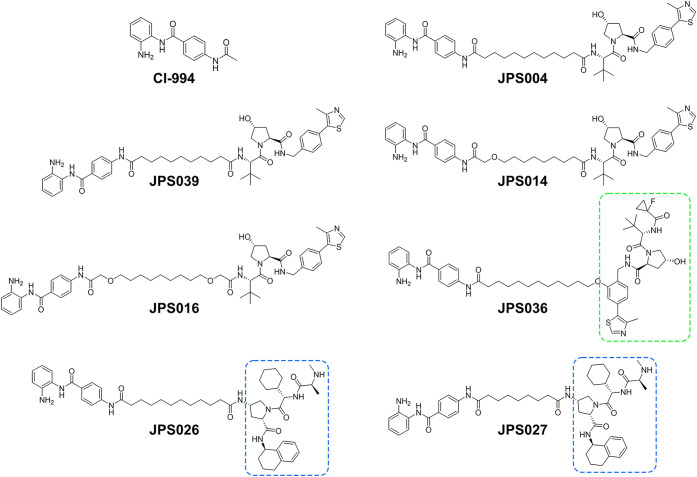
Schematic representation of the class I HDAC targeting PROTAC library used during this study. PROTACs JPS004, JPS014, JPS016, JPS039, and JPS036 incorporate the von Hippel-Lindau E3 ubiquitin ligase ligand, while PROTACs JPS026 and JPS027 incorporate the inhibitor of apoptosis protein (IAP) E3 ubiquitin ligase ligand.

CI-994 is a class I HDAC inhibitor that chelates zinc in the HDAC active site through coordination with the *o*-amino anilide functional group, with *K*_i_ values reported for HDAC1 and HDAC3 of 0.41 and 0.75 μM, respectively.^17^ Other CI-994 analogues currently in clinical trials include entinostat (MS-275) and mocetinostat (MGCD0103),^18^ while chidamide (HBI-8000) has been approved by the Chinese FDA for the treatment of peripheral T cell lymphoma.^31^ We have derived a number of PROTACs that utilize the zinc-chelating *o*-amino anilide group present in CI-994, such as the previously reported JPS004, which contains a 12-atom alkyl linker and a VHL E3 ligase ligand, capable of degrading HDAC1/2 and HDAC3 in HCT116 cells.^21^ We also recently reported an updated PROTAC library that includes new PROTACs JPS014, JPS016, JPS036, and JPS039 (shown in Figure 1), incorporating various modifications to either the alkyl linker or VHL E3 ligase ligand used. We found that variations in linker length and the incorporation of oxygen atoms afforded submicromolar DC_50_ values for HDAC1 and HDAC3, while the addition of a fluoro-cyclopropyl group to the VHL ligand of JPS036 surprisingly revealed selectivity toward HDAC3.^22^ To further extend the portfolio of CI-994-based degraders and examine the effectiveness of alternate E3 ligase ligands, we synthesized novel PROTACs JPS026 and JPS027 (Figure 1, screened in Figures S4 and S5) that incorporate a ligand for the inhibitor of the apoptosis protein (IAP) family of E3 ligases, which has previously been used in degraders of Bruton’s tyrosine kinase (BTK^32,33^). In these initial designs, JPS026 can be described as an IAP analogue of JPS004 including the 12-atom linker, while JPS027 includes a shorter nine-atom linker (Figure 1; supporting chemistry and characterization for JPS026 and JPS027 can be found in the Supporting Information).

### A Novel Combination of HDAC and IAP Ligands Generates a PROTAC with Potent Ability to Stimulate Apoptosis in Colon Cancer Cells

**Figure 2:**
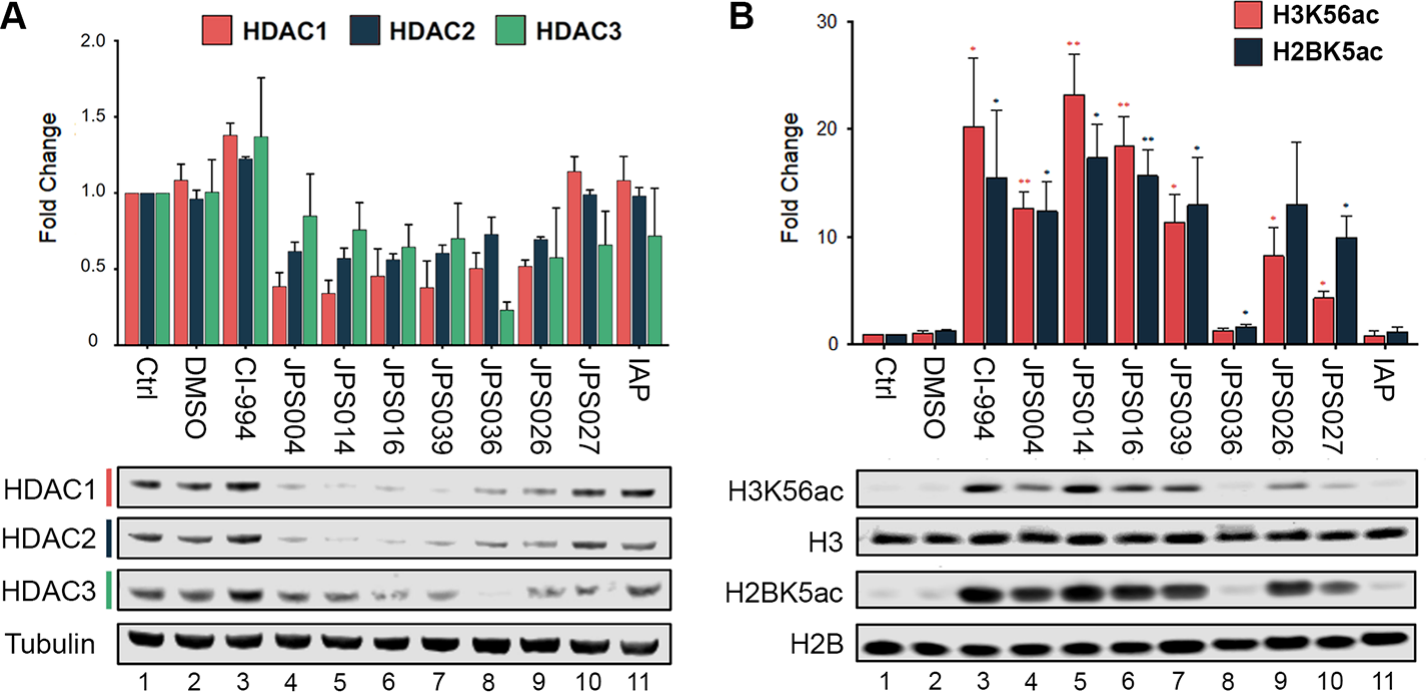
Quantitative Western blotting reveals that PROTAC-mediated degradation of class I HDACS, HDAC1–3, leads to an increase in the levels of H3K56 and H2BK5 acetylation in colon cancer cells. HCT116 cells were treated VHL-based PROTACs (JPS004, JPS014, JPS016, JPS039, and JPS036 dosed at 10 μM) or IAP-based PROTACs (JPS026 and JPS027 dosed at 5 μM), with relevant controls (DMSO, 10 μM CI-994, and 5 μM IAP) for 24 h before analysis via quantitative Western blotting and normalization of protein levels to relevant controls. (A) Protein levels of HDAC1–3 in HCT116 cells after PROTAC treatment. (B) Statistical analysis of levels of acetyl-histone H3 at lysine 56 (H3K56Ac) and acetyl-histone H2B at lysine 5 (H2BK5Ac) in HCT116 cells, two histone marks associated with actively transcribed genes. Data are presented as the mean ± standard deviation of three independent biological replicates. *p ≤ 0.05, and **p ≤ 0.01.

HCT116 cells were treated with the novel PROTACs for 24 h, and then the levels of HDAC1–3 were assessed by quantitative Western blotting. Untreated and DMSO controls showed baseline levels of all three proteins, with CI-994 treatment producing a small but consistent increase in the level of each of the HDACs (in Figure 2A, compare lanes 1–3). Consistent with previous reports, PROTACs JPS004, JPS014, and JPS016 all induced degradation of HDAC1–3 (Figure 2A, lanes 4–6, respectively). There is a slight preference for HDAC1/2 degradation over HDAC3 with these compounds, possibly due to the greater abundance of HDAC1/2, which can thus outcompete HDAC3 for the PROTAC.^22^ JPS039, identical to JPS004 but with an 11-atom alkyl linker, behaves in a manner almost identical to that of its sister molecules. Initially, JPS026 was added to cells at a concentration of 10 μM, similar to the other PROTACs tested, but it was found that a 24 h treatment at this concentration caused almost complete cell death, making downstream analysis of proteins and RNA unfeasible. Therefore, PROTACs incorporating the IAP ligand were restricted to 5 μM treatments in subsequent experiments. JPS026 at 5 μM produced levels of HDAC1–3 degradation similar to those seen with JPS004 (Figure 2A). However, as with previous attempts to modify the linker length,^21^ a decrease to nine atoms in JPS027 caused a loss of HDAC degradation, while the IAP ligand alone has relatively little effect (in Figure 2B, compare lanes 9–11).

To test the ability of PROTACs to penetrate cells and inhibit class I HDACs, we measured lysine acetylation levels in histone tails. We examined two independent sites of acetylation, Lys56 on histone H3 (H3K56ac), a known HDAC1/2 substrate,^34^ and Lys5 on histone H2B (H2BK5ac), a mark of active transcription,^35^ by quantitative Western blotting (Figure 2B). Treatment of cells with CI-994 produced robust 20- and 15-fold increases in H3K56ac and H2BK5ac levels, respectively, compared to controls. JPS004 consistently produced an increase that was smaller than that of CI-994 (in Figure 2B, compare lanes 3 and 4), while acetylation levels in the presence of JPS014 and JPS016 were similar to those of CI-994, demonstrating the advantage of introducing an oxygen atom into the alkyl linker. Interestingly, JPS036, the PROTAC with specificity toward HDAC3, showed relatively little change in histone acetylation, indicating that HDAC1/2-containing complexes are the major regulators of these sites in HCT116 cells. Treating cells with the IAP-based PROTACs, JPS026 and JPS027, generated increased levels of both H3K56ac and H2BK5ac, although the latter was relatively modest compared to those of other PROTACs tested (Figure 2B). Again, JPS026 outperformed JPS027, confirming that a 12-atom linker is preferable to a nine-atom linker under these conditions. As expected, the IAP ligand alone had no effect on histone acetylation compared to controls (in Figure 2B, compare lanes 1, 2, and 11).

**Figure 3:**
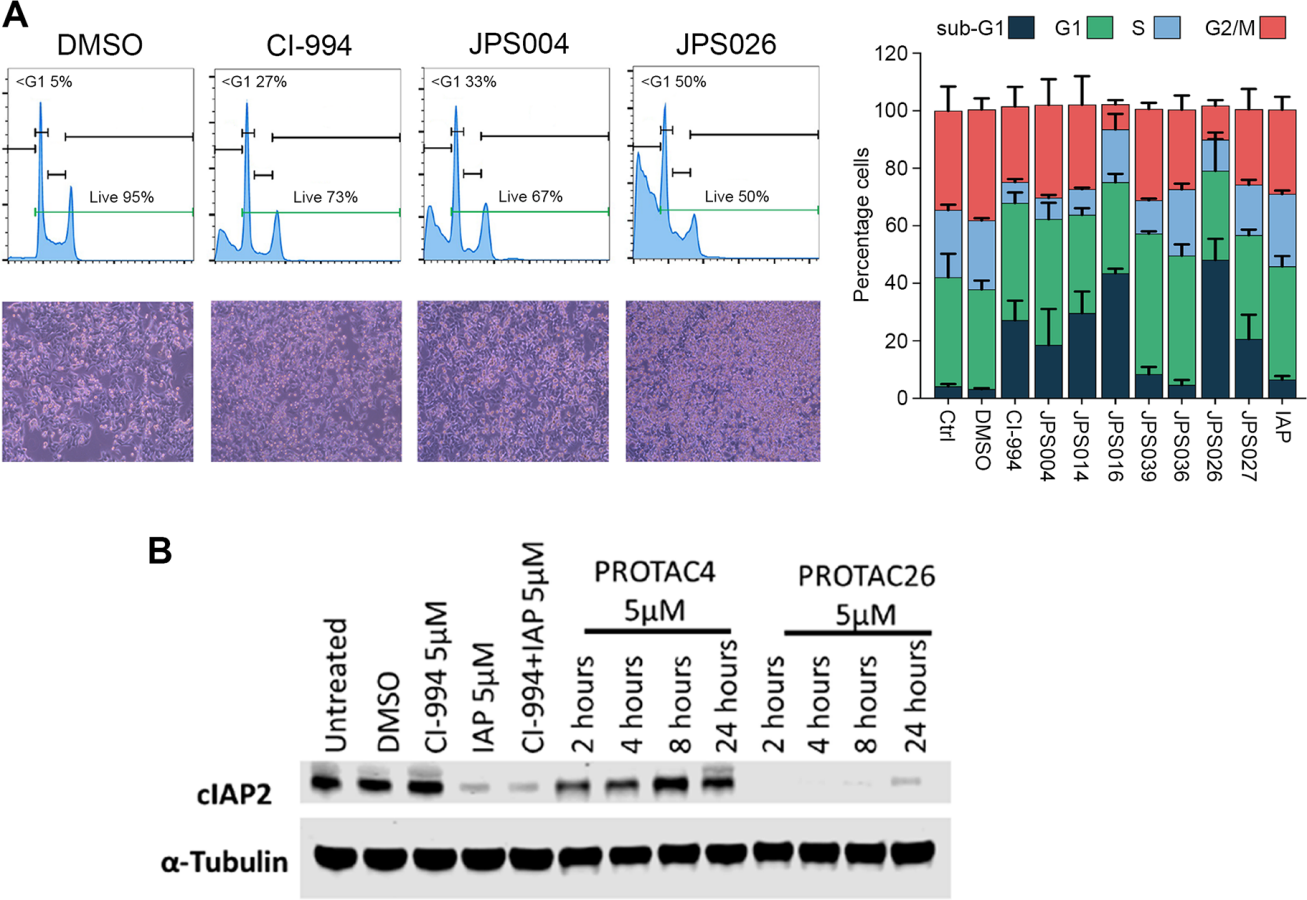
Viability and cell cycle distribution of colon cancer cells upon treatment with PROTACs targeting class I HDACs. (A) HCT116 cells were treated for 24 h with PROTACs containing modifications to either the alkyl linker (JPS004, JPS014, JPS016, JPS039, and JPS039 dosed at 10 μM) or ubiquitin E3 ligase ligand (JPS026 and JPS027 dosed at 5 μM) before being stained with propidium iodide (PI) and analyzed by flow cytometry. The left panel shows representative histograms depicting the cell cycle distribution with accompanying cell images (20× magnification) of HCT116 cells treated with DMSO, CI-994, JPS004, and JPS026. The right panel shows a stacked bar graph of the cell cycle distribution. Data are presented as the mean ± standard deviation of three independent biological replicates. (B) Western blot showing the levels of cIAP2 protein in HCT116 cells following the indicated treatments.

Benzamide-based HDACi have a well-known capacity to induce apoptosis in cancer cells and have been tested in a range of tumor types.^17^ The functionalization of CI-994 into an HDAC1/2 and 3 degrader, using a standard VHL ligand (JPS014 and JPS016), improved its ability to induce cell death in HCT116 cells (Figure 3A). We were able to assess the contribution using different E3 ligase ligands by comparing JPS004 and JPS026, because both contain the same 12-atom alkyl linker and HDAC ligand, differing only in their respective E3 ligase ligands. JPS026, the IAP-containing PROTAC, produced almost 2-fold more cell death than JPS004 did (Figure 3A, right panel, average of 27% vs 50% sub-G1 cells), despite being used at a concentration of only 5 μM. In contrast, the IAP ligand alone showed no significant alteration in cell cycle or cell death in HCT116 cells, although it is known to induce cell death in other cell types. The IAP ligand, alone or incorporated into a PROTAC,^32,36^ is capable of causing autoubiquitination of the E3 ligase itself, leading to a loss of protein. To assess whether this was the case with JPS026, we treated HCT116 cells with JPS004 and JPS026 and performed a Western blot for cIAP2 levels (Figure 3B). The IAP ligand alone, in combination with CI-994 or JPS026, caused a downregulation of cIAP2 levels compared to controls (DMSO and JPS004), suggesting that autoubiquitination is also responsible for the loss of cIAP2 observed here. However, in HCT116 cells at least, treatment with an IAP ligand alone caused relatively little cell death. Thus, the combination of CI-994 and an IAP E3 ligase ligand, with an optimized linker, is a novel and potent inducer of apoptosis in colon cancer cells.

### A Comprehensive Transcriptomic Analysis of HCT116 Cells Treated with CI-994 and PROTACs Reveals Significant Changes in HDAC Complex Components

Class I HDACs regulate histone acetylation across the genome, and their inhibition leads to widespread changes in gene expression.^37^ Despite a range of HDACi being tested on different cancer cell types, as well as their use in the clinic, there is still no consensus about their mode of action in stimulating cell cycle arrest and cell death. There is, however, a widely held assumption that changes in gene expression lie at the heart of this activity. For instance, a robust increase in the cell cycle inhibitor, p21, is observed with the application of many HDACi.^38^ However, p21 is the tip of the iceberg with many thousands of genes being both up- and downregulated by HDAC inhibition. Furthermore, many cancer cells have a deregulated G1/S transition at which inhibition DEP domain-containing mTOR interacting protein (DEPTOR) of cyclin D/CDK4/6 is unlikely to be a major factor. Given the number of novel HDAC1/2 and -3 degraders within our PROTAC library, we decided to pursue a comprehensive investigation of their effects on gene expression in parallel.

**Figure 4:**
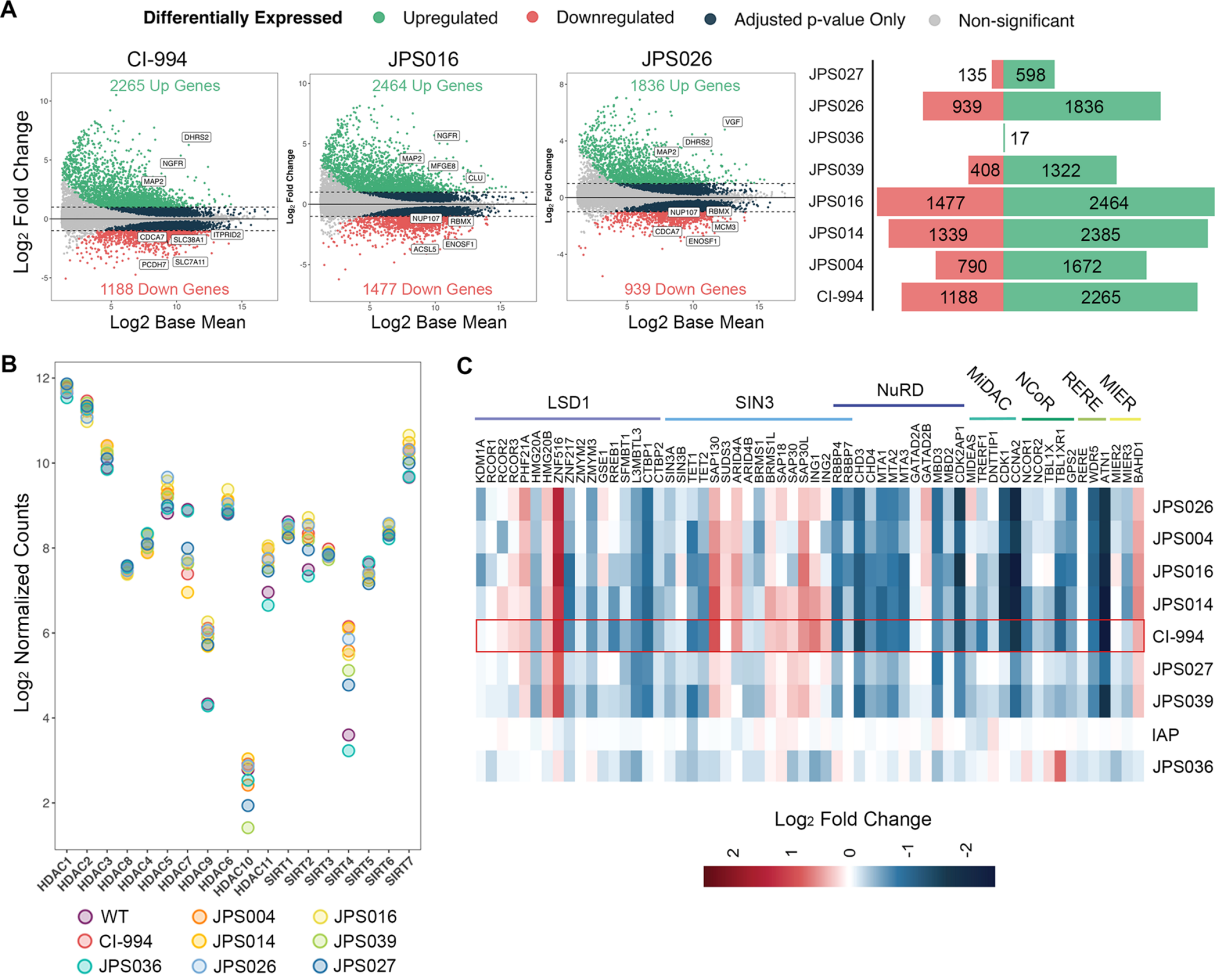
RNA sequencing analysis reveals profound transcriptional defects in colon cancer cells treated with class I HDAC targeting PROTACs. (A) MA plots (left panel) and bar graph (right panel) showing summarized totals of up- and downregulated differentially expressed genes (DEGs) in HCT116 cells treated with VHL-based PROTACs (JPS004, JPS014, JPS016, JPS036, and JPS039 dosed at 10 μM) or IAP-based PROTACs (JPS026 and JPS027 dosed at 5 μM). DEGs were defined as genes displaying a *p*-adjusted value of <0.01 and a log fold change of more than ±2 (log_2_ fold change > ±1). (B) Normalized counts of HDAC genes. Data are presented as log_2_ normalized counts. (C) Heat map displaying log_2_ fold changes in gene expression of HDAC co-repressor complexes.

HCT116 cells were treated with either DMSO, CI994, the seven different PROTACs studied here (JPS004, JPS014, JPS016, JPS026, JPS027, JPS036, and JPS039), or the IAP ligand alone for 24 h before RNA was extracted for RNA-seq analysis. Our study consisted of 27 individual experiments (three biological replicates across nine different conditions), adding a statistical robustness and weighting to the differentially expressed genes observed. Principal component analysis (PCA) revealed robust clustering between replicates for individual treatments and that structurally related compounds (e.g., JPS004, JPS014, and JPS016) also clustered closely to each other (Figure S1A). There was a strong correlation between the ability of a compound to increase histone acetylation levels (Figure 2B) and the number of DEGs (Figure 4A and Figure S2). For example, JPS036, which showed specificity toward HDAC3 (Figure 2A), produced only modest increases in H3K56ac levels and just 17 DEGs (Figure 4A). JPS016, in contrast, produced a 20-fold increase in the H3K56ac level and a total of 3941 DEGs. JPS026, the more effective of the two IAP PROTACs, showed 1836 genes upregulated and 939 genes downregulated (2775 DEGs total). This was fewer than the number for parental inhibitor CI-994 but similar to the number for its counterpart, JPS004 [2462 DEGs, of which 2051 (74%) were overlapping (Figure S1B)]. Treatment of HCT116 cells with the IAP ligand alone did not produce any differential gene expression (Figure S2).

We next examined the expression of the 18 different HDAC enzymes expressed in HCT116 cells (Figure 4B). It was possible that cells might compensate for the loss of HDAC activity, particularly following HDAC1/2 and -3 protein degradation. Surprisingly, there was little change in HDAC1–3 expression following CI-994 or PROTAC treatment. There were, however, significant changes in the expression of other HDACs, suggesting a shift in acetylome regulation following the loss of the class I HDACs. Among the class IIa and IV HDACs, we observed a decrease in HDAC7 levels with a compensatory increase in HDAC9 and HDAC11 levels. Sirtuins are NAD-dependent HDACs (class III) with a distinct enzymatic domain and mode of catalysis.^37^ Here we found that there were also relatively modest changes, with only SIRT4 and SIRT7 levels increasing 4- and 2-fold, respectively.

Class I HDACs (with the exception of HDAC8) exist as central components of numerous multiprotein complexes in cells.^8^ Knockout studies of HDAC1/2 have shown that removing the enzymes from the complex leads to an increased rate of turnover of the remaining proteins, indicating a structural role.^15,16^ Again, it seemed probable that there might be compensation for the loss of complex integrity following PROTAC-mediated degradation. We therefore examined the expression of the various components of the seven major class I HDAC complexes (Figure 4C). Intriguingly, we found significant decreases in nearly all of the core components of the nucleosome remodeling and deacetylase (NuRD) complex, including all three scaffold paralogs, MTA1–3, MBD3, and the ATP-dependent helicase, CHD4. MiDAC (mitotic deacetylase complex) is an atypical HDAC1/2 complex, originally described as mitotic specific, and contains cell cycle regulators CDK1 and cyclin A2, which are both downregulated >4-fold following PROTAC treatment. However, the core trio of DNTTIP1, MIDEAS, and HDAC1 is unaffected by HDAC inhibition, suggesting the core of the MiDAC complex is unaltered. Several components of the Sin3 complex are upregulated, including SAP130, SUDS3, ARID4A, and SAP30L, although intriguingly there are modest reductions in the level of the complex backbone, SIN3A (Figure 4C). These altered gene expression patterns were shared among both VHL- and IAP-derived PROTACs (in Figure 4C, compare JPS004 and JPS026), suggesting their activity was independent of the E3 ligase ligand used. Indeed, many of these changes occur with both CI-994 and PROTACs, suggesting that HDAC inhibition is the main driver of the altered expression. Finally, although the HDAC3 specific PROTAC JPS036 showed very limited changes in gene expression, it was notable that NCoR1, TBL1X, and TBL1XR1 were all upregulated, a signature quite distinct from those of the other PROTACs used in this study and seemingly confirming its selectivity toward the NCoR/HDAC3 complex.

### HDAC Inhibition and Degradation Cause a Dramatic Decrease in Cell Cycle and DNA Replication Machinery

**Figure 5:**
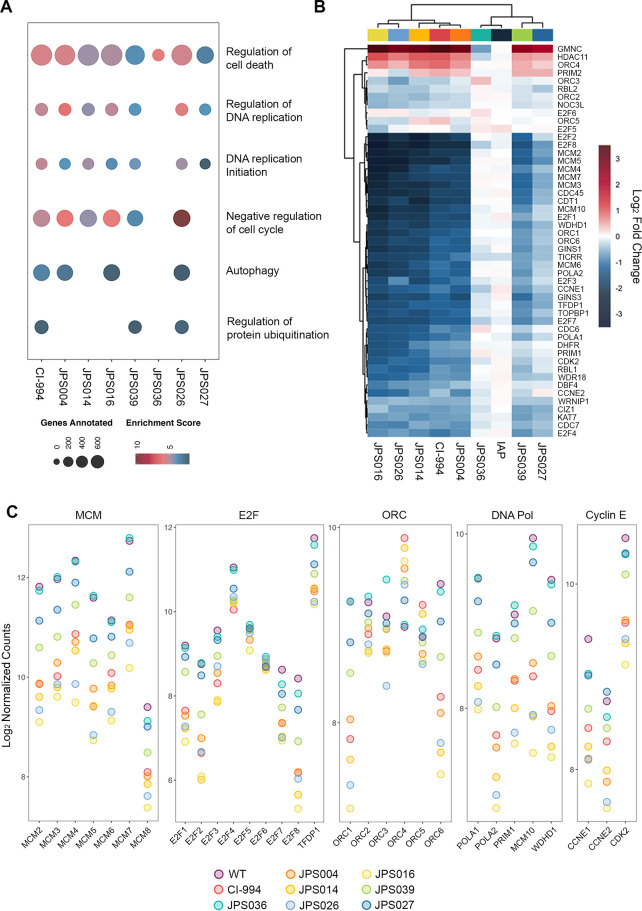
Gene ontology analysis shows enrichment of biological processes, including the regulation and initiation of DNA replication upon exposure of colon cancer cells to PROTACs. HCT116 cells were subjected to RNA sequencing analysis after 24 h treatments with VHL ligand-containing PROTACs (JPS004, JPS014, JPS016, JPS036, and JPS039 dosed at 10 μM) or IAP ligand-containing PROTACs (JPS026 and JPS027 dosed at 5 μM). (A) Enriched gene ontology terms identified from differentially expressed gene lists arising from PROTAC-treated HCT116 cells. Enrichment is defined as −log 10 of the *p*-adjusted value, and differentially expressed genes (DEGs) were classified as genes displaying a *p*-adjusted value of <0.01 and a log fold change of more than ±2 (log_2_ fold change > ±1). (B) Heat map displaying log_2_ fold changes in gene expression of key genes involved in DNA replication. (C) Normalized counts of gene families involved in DNA replication. Data are presented as log_2_ normalized counts.

Gene ontology (GO) analysis of the DEGs produced by PROTAC treatment of HCT116 cells revealed notable enrichment of GO terms involved in biological processes, including changes in DNA replication, negative regulation of the cell cycle, and cell death (Figure 5A). JPS036 served as a useful negative control because it produced only 17 DEGs in total. A heat map containing a manually curated list of cell cycle regulators confirms the strong antiproliferative phenotype of CI-994 and PROTACs, particularly JPS014, JPS016, and JPS026 (Figure 5B, left three columns). While all PROTACs showed downregulation of cell cycle genes, we again observed that longer linker lengths (JPS004 vs JPS039 and JPS026 vs JPS027) produced a stronger effect on gene expression. We also examined the normalized read counts for related families of cell cycle machinery in all treatments (Figure 5C). The pre-replicative, heterohexamic complex composed of the minichromosomal maintenance complex component 2–7 (MCM2–7, respectively) proteins helps to both initiate DNA replication and stimulate elongation via its helicase activity. All six components are downregulated upon PROTAC treatment in HCT116 cells (Figure 5B,C). Compellingly, the reduction is dependent upon the effectiveness of the PROTAC used: JPS016 > JPS026 > JPS004 > JPS039 > JPS036 in all genes examined. A number of factors that positively regulate the G1/S transition, including E2F1–5, cyclin E1 (CCNE1), and cyclin-dependent kinase 2 (CDK2), were similarly reduced by CI-994 and class I HDAC degraders. Both catalytic (POLA1) and regulatory (POLA2) subunits of DNA polymerase were also significantly reduced. Loss of HDAC1/2 activity (but, possibly not HDAC3 resulting from JPS036 treatment) produces a comprehensive reduction in the cell cycle apparatus that is dependent upon the effectiveness of the inhibitor demonstrated across a range of activities.

The enrichment of GO terms relating to cell death was unsurprising because we observed significant increases in the sub-G1 population of cells with CI-994 and PROTAC treatments (Figure 3A). GO analysis also identified a modest enrichment in the regulation of protein ubiquitination, a relevant term in the context of this system given the mode of action of PROTACs. Upon further analysis, it was found that there was a noteworthy upregulation in genes encoding non-ATPase 8 and 9 (PSMD8/9) subunits of the 26S proteasome. A modest increase in the level of gene expression of ubiquitin B and C (UBB and UBC, respectively) was also observed for the two most potent PROTACs in the library, JPS016 and JPS026 (Figure S1C).

### Treatment of HCT116 Cells with CI-994 and PROTACs Leads to Transcriptional Changes in the AKT1/mTOR Signaling Pathway

**Figure 6:**
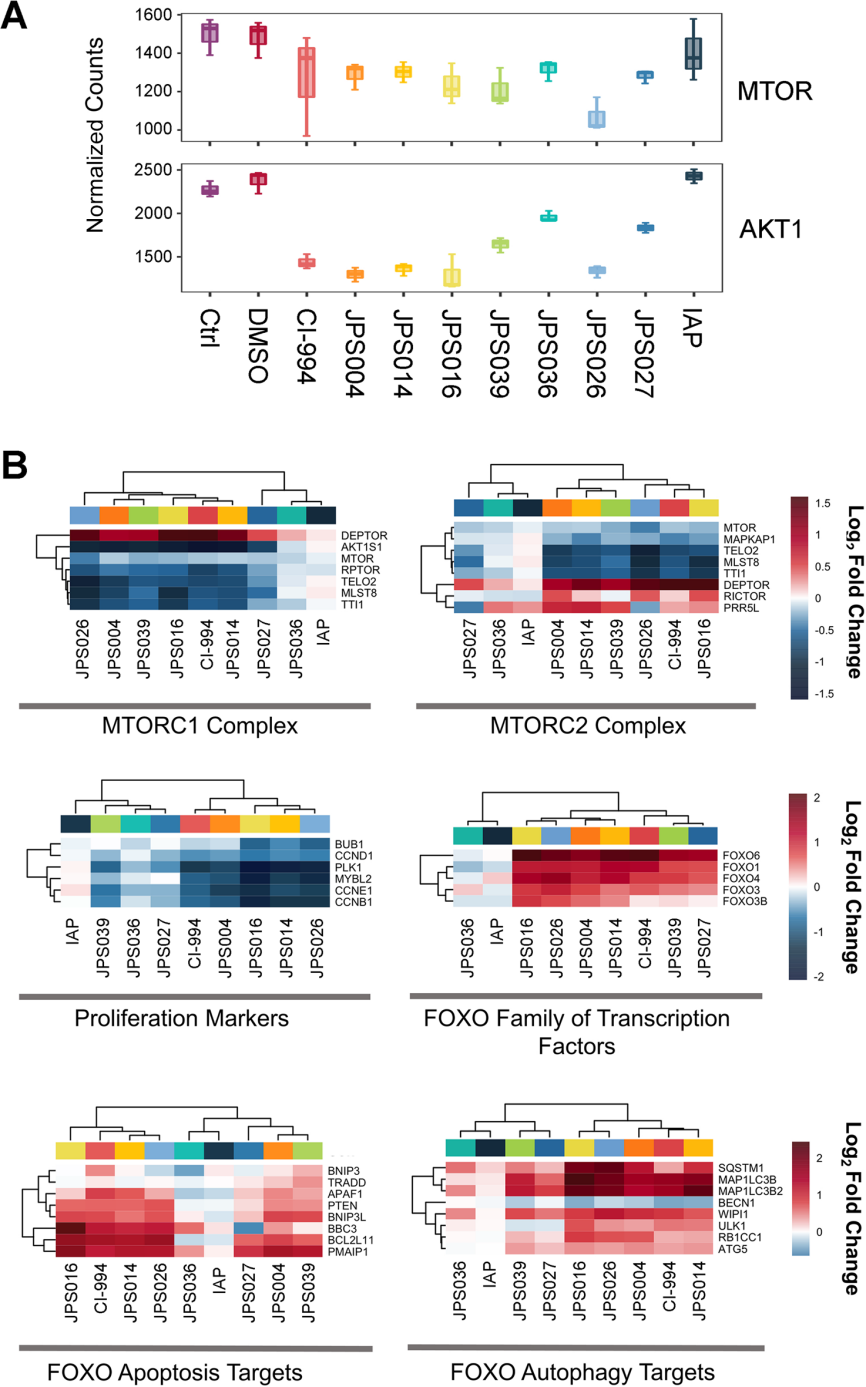
Transcriptional analysis of PROTAC-treated colon cancer cells reveals downregulation of the mTOR signaling pathway and upregulation of downstream FOXO transcription factors and autophagy-related genes. HCT116 cells were exposed to VHL-based PROTACs (JPS004, JPS014, JPS016, JPS039, and JPS036 dosed at 10 μM) or IAP-based PROTACs (JPS026 and JPS027 dosed at 5 μM) for 24 h before gene expression was analyzed through RNA sequencing. (A) Box plots of AKT1 and MTOR normalized counts. (B) Heat maps of protein families involved in the AKT/mTOR signaling pathway and downstream regulation of autophagy. Heat maps are presented as the average log_2_ fold changes across three independent biological replicates.

The AKT serine/threonine kinase 1 (AKT1) and mammalian target of rapamycin (mTOR) signaling axis is widely known for its involvement in numerous biological processes, notably cell proliferation and survival.^39,40^ mTOR operates as the catalytic subunit of multiprotein mTOR complexes 1 and 2 (mTORC1 and mTORC2, respectively), playing roles in the downstream inhibition of the forkhead box O (FOXO) family of transcription factors and thereby influencing expression of FOXO3 target genes pertaining to biological processes such as autophagy, cellular atrophy, and apoptosis.^41−43^ We observed a small but consistent decrease in AKT1 and MTOR expression in HCT116 cells treated with CI-994, JPS004, JPS014, JPS016, and JPS026 (Figure 6A). The more notable transcriptional changes in the pathway occurred to the mTORC1 and mTORC2 complex members and their downstream targets. Both CI-994 and the more potent PROTACs (e.g., JPS016 and JPS026) led to downregulation of the shared genes encoding the mTORC1/2 complex: mammalian lethal with Sec-13 (MLST8), TELO2, and TTI1 (Figure 6B). However, transcription of the natural mTORC1/2 inhibitor, DEP domain-containing mTOR-interacting protein (DEPTOR) was significantly upregulated, despite the presence of a negative feedback loop.^44^ This suggests a downregulation in the activity and expression of the mTORC1/2 complexes. Consistent with transcriptional repression of AKT1 and MTORC1/2, we observed a downregulation of key proliferation markers such as CCNB1 (cyclin B1) and MYBL2 and upregulation of FOXO transcription factors (Figure 6B). Most notably, increases in the level of expression of FOXO3 are responsible for the transcriptional induction of both apoptosis and autophagy through the transcription of FOXO3 target genes, including BCL2L11 (Bim), PMAIP1 (NOXA), and BBC3 (PUMA) involved in the induction of apoptosis as well as ULK1, RB1CC1, and SQSTM1, which are key markers of autophagy.^41,45^ The upregulation of FOXO3 paired with the enrichment of autophagy (Figure 5A) and previously discussed apoptosis (Figure 3A and ref (21)) supports an induction of cell death partly driven through the transcriptional changes induced by PROTAC and HDACi treatments.

## Discussion

We have generated a novel library of class I HDAC degraders, including the first PROTAC directed against HDAC1/2 that uses a ligand to the IAP family of E3 ligases (JPS026 and JPS027). Both VHL- and IAP-based PROTACs led to equivalent degradation of HDAC1/2 and HDAC3 (in Figure 2A, compare JPS004 with JPS026). However, we found that HCT116 cells were far more sensitive to JPS026 than to JPS004, with at least 2-fold more cell death even at decreased concentrations (Figure 3A). Intriguingly, JPS026 treatment produced approximately the same increase in H3K56ac levels (Figure 2B) and differential gene expression levels (Figure 4A) as JPS004, suggesting that its improved ability to induce cell death occurs in addition to its HDACi activity. Other PROTACs generated using the same IAP ligand, such as the degraders of BTK described by Tinworth et al., remove both the protein of interest and the E3 ligase itself.^32^ To examine whether this was also the case with JPS026, we blotted for cIAP2 and found that it was rapidly degraded in HCT116 cells following treatment with JPS026 but, importantly, not with JPS004, which contains a VHL ligand (Figure 3B). In contrast, cellular protein levels of VHL have previously been shown to remain unchanged upon PROTAC treatment with VHL ligand-based PROTACs targeted against Cereblon in multiple cell types.^46,47^ Despite being largely cytoplasmic proteins, members of the the IAP family (cIAP1, cIAP2, and XIAP) have been used in PROTACs that target a number of nuclear proteins for degradation, including the estrogen and androgen receptors (reviewed in ref (33)).

We have shown that PROTACs with short linkers are better HDAC inhibitors *in vitro* than longer linkers but that the opposite is true in cells.^21^ Here again, we found that increases in the level of histone acetylation and the number and degree of differentially expressed genes were greater with longer linkers (≥11 atoms; JPS004 > JPS039 and JPS026 > JPS027). Despite the higher molecular weight, surprisingly this seems to argue that the longer linker allows for greater cell permeability of the PROTAC, in defiance of Lipinski’s rule of five.^48^ In terms of gene regulation, there was no significant difference between inhibition (CI-994) and inhibition plus degradation [i.e., PROTACs (see Figure 4A)]. JPS016 appears to be marginally more effective than CI-994 at inducing apoptosis (Figure 3A) and perturbing gene expression (Figure 4A). However, the gene signatures produced by both molecules were largely the same, with notable decreases in cell cycle and DNA replication machinery (Figure 5B). A strength of this study is the use of multiple PROTACs in parallel, allowing us to assign DEGs with greater confidence and revealing a dose-dependent effect based on their potency to increase the level of histone acetylation: JPS016 > JPS026 > JPS004 > JPS039 > JPS036 (Figures 2B and 5C). A 24 h treatment was sufficient to induce significant decreases in DNA replication machinery, including the MCM2–7 and ORC complexes, as well as DNA polymerase subunits (Figure 5C). Our data are also consistent with previous transcriptomic studies exploring the effects of treatment of HCT116 cells with HDACi. Microarray data obtained by LaBonte et al.,^49^ using LBH589, a pan-inhibitor for zinc-dependent HDACs, also showed downregulation of cell cycle-related genes, including cyclin A2, E2F2, and CDCA7, in line with our findings for CI-994 and PROTAC (JPS004, JPS1016, and JPS026) treatments. Furthermore, Liu et al.,^50^ reported microarray data displaying an upregulation in the cell cycle inhibitor CDKN1A (p21) and pro-apoptotic BCL-2-like protein 11 (BCL2L11), also known as BIM, which we also observed to be upregulated upon CI-994 and PROTAC treatment (JPS016 and JPS026), thus suggesting a core set of regulatory genes contribute to a shared mechanism of action that is responsible for the cell cycle- and cell death-related phenotypes observed in cells treated with HDACi.

Aberrant AKT/mTOR signaling is a hallmark observed in many cancer types, including colon cancer, angiosarcoma, and breast cancer.^51−53^ The synergistic interplay between HDACi and AKT/mTOR signaling has also been proposed as a potential mechanism of action behind the anticancer effects of HDACi in a variety of cancer models (reviewed in refs (54) and (55)). However, these studies by and large have been performed using pan-HDAC inhibitors (e.g., TSA, SAHA, etc.) that lack the class I HDAC specificity of the PROTACs used in our study. We have shown that HDAC degradation and inhibition exert a notable effect on the transcriptional regulation of the AKT/mTOR/FOXO signaling axis and cell death above those observed for CI-994 treatment alone (Figures 3A and 6A,B). Therefore, the ability to transcriptionally target both of these aberrant phenotypes and induce cell death in colon cancer cells through the use of single class I HDAC inhibitors and novel degraders, such as PROTACs described here, offers an exciting potential avenue for the clinical treatment of colon cancer.

## Supporting Information Available

Chemical synthesis and characterization of PROTACs JPS026 and JPS027 and additional supporting biological data and antibody information (PDF)

## Abbreviations

H2BK5ac: acetylation of Lys5 on histone H2B
H3K56ac: acetylation of Lys56 on histone H3
AKT1: AKT serine/threonine kinase 1
BTK: Bruton’s tyrosine kinase
CoREST: co-repressor of REST
CDK2: cyclin-dependent kinase 2
DEPTOR: DEP domain-containing mTOR-interacting protein
DEG: differentially expressed gene
FOXO: forkhead box O
GO: gene ontology
HDAC: histone deacetylase
HDACi: HDAC inhibitors
IAP: inhibitor of apoptosis protein
MLST8: mammalian lethal with Sec-13
mTOR: mammalian target of rapamycin
MCM: minichromosomal maintenance complex component
mTORC: mTOR complex
Kac: ne-lysine acetylation
PSMD: non-ATPase
NuRD: nucleosome remodeling and deacetylase
PCA: principal component analysis
PI: propidium iodide
PROTAC: proteolysis targeting chimera
SIRT: sirtuin
Sin3: switch-independent protein 3
VHL: von Hippel-Lindau.
