# Supplementary material for: Comprehensive Transcriptomic Analysis of Novel Class I HDAC Proteolysis Targeting Chimeras (PROTACs)
Source: Biochemistry. 2022 Aug 10;62(3):645–56. doi: 10.1021/acs.biochem.2c00288 (PMC9910044; doi:10.1021/acs.biochem.2c00288)

Supplemental Information

Comprehensive transcriptomic analysis of novel class-I HDAC degraders

OR

PROTAC Mediated Degradation of Class I HDACs Leads to Cell Death and Profound Transcriptional Defects in Colon Cancer Cells

India M. Baker,[a] *Joshua P. Smalley,*[b] *Khadija A. Sabat,*[a] *James T. Hodgkinson*[b*] *and Shaun M. Cowley*[a*]

a. Department of Molecular and Cell Biology, University of Leicester, Leicester, LE1 7RH, UK

b. Leicester Institute of Structural and Chemical Biology, School of Chemistry, University of Leicester, Leicester, LE1 7RH, UK

Table of Contents

SUPPLEMENTAL INFORMATION: BIOLOGY 3

1. RNA-SEQUENCING 3
2. FLOW CYTOMETRY 4
3. ANTIBODIES USED FOR QUANTITATIVE WESTERN BLOTTING 5
4. WESTERN BLOTTING - INITIAL SCREEN OF JPS026, JPS027 AND IAP LIGAND 5

SUPPORTING INFORMATION: CHEMISTRY 7

1. MATERIALS AND METHODS 7
2. PREPARATION OF JPS026 AND JPS027 8
3. PREPARATION OF IAP LIGAND 12

APPENDIX: ^{1}H NMR AND ^{13}C NMR OF NOVEL COMPOUNDS 14

Supplemental Information: Biology

1. RNA-sequencing

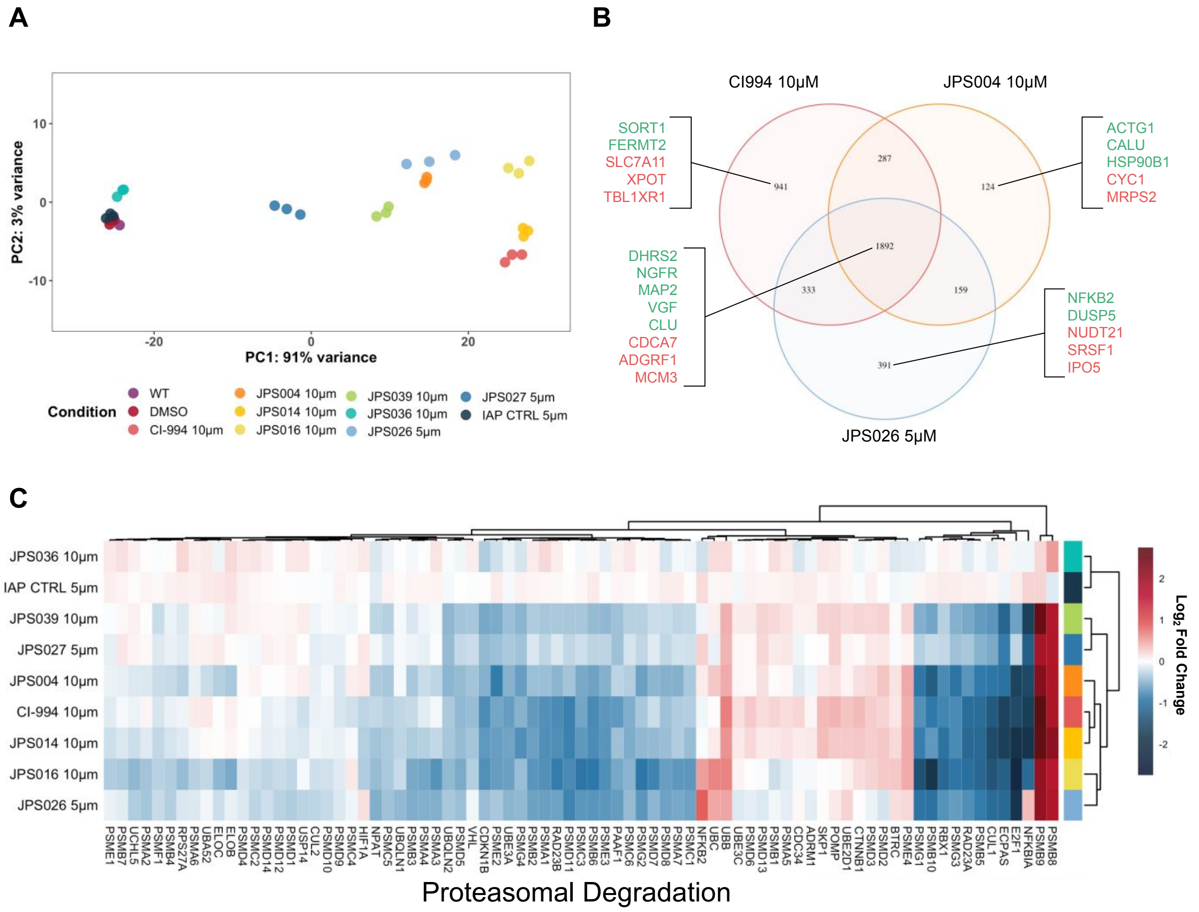

Figure S1: **RNA-sequencing of HCT116 cells treated with PROTAC library targeting Class I HDACS.** HCT116 cells were treated with VHL-based PROTACs (JPS004, JPS014, JPS016, JPS039, and JPS036 dosed at 10 µM) or IAP based PROTACs (JPS026, JPS027 dosed at 5 µM) for 24 hours and subjected to RNA-sequencing analysis. (A) Principal component analysis (PCA) score plot of sample clustering. (B) Overlap in differential gene expression between CI-994 (parent inhibitor), JPS004 (VHL analogue), and JPS026 (IAP ligand analogue). Differentially expressed genes were defined as genes displaying a p-adjusted value < 0.01 and a log fold change $> \pm 2$ (log2 fold change $> \pm 1$. (C) Heatmap displaying log2 fold changes in expression of a manually comprised gene list relating to proteasomal degradation.

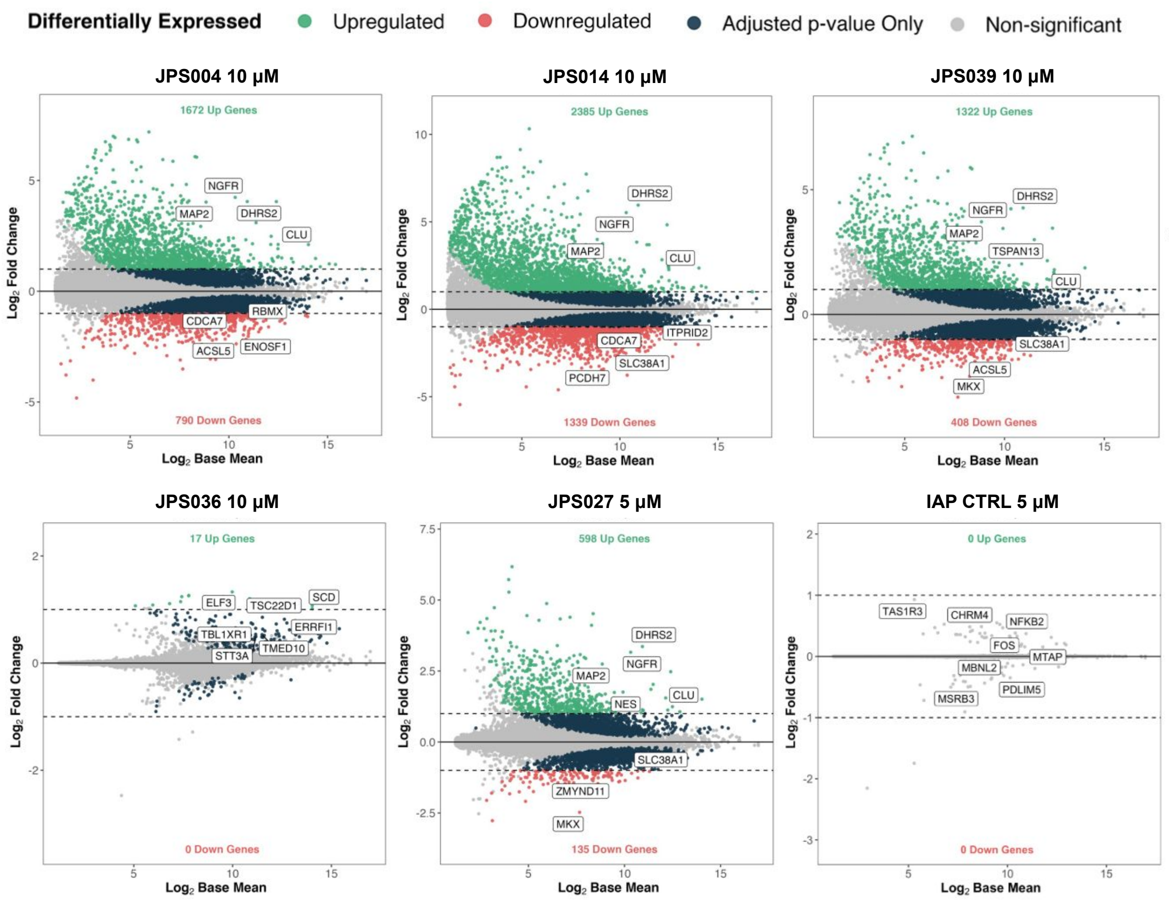

Figure S2: **Transcriptomic profiles of HCT116 cells treated with HDAC1/2/3 targeted PROTACs.** HCT116 cells were treated with VHL-based PROTACs (JPS004, JPS014, JPS039, and JPS036 dosed at 10 μM) or IAP based PROTACs (JPS026, JPS027 dosed at 5 μM). Differential gene analysis was performed in DESeq2 and presented as MA plots. Differentially expressed genes were defined as genes with a significance p-adjusted <0.01 and a $\log_2$ fold-change > ± 1.

2. Flow cytometry

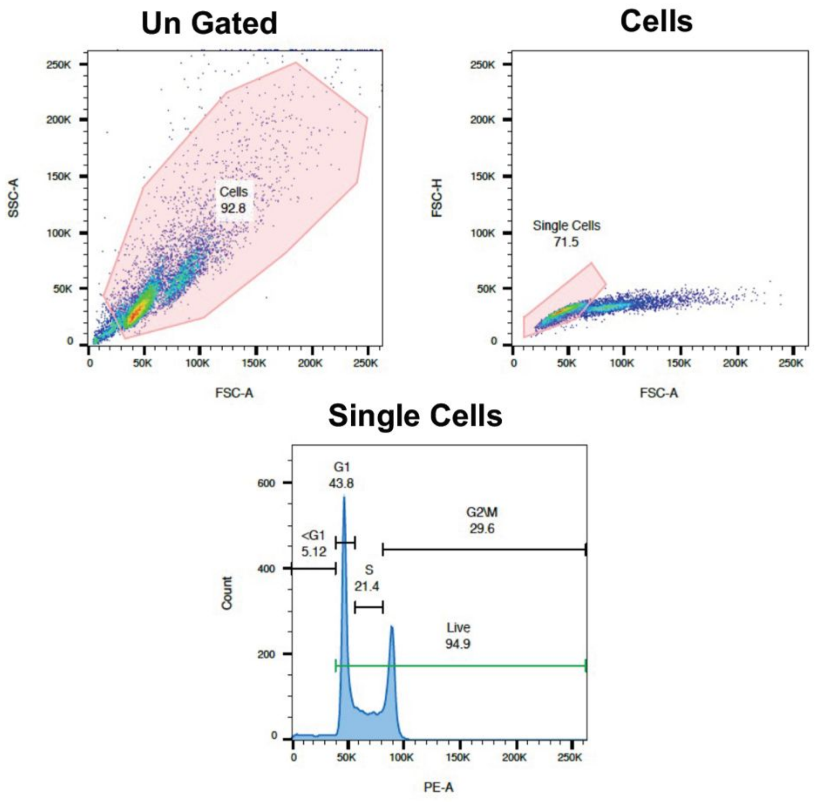

Figure S3: **Gating strategy applied to HCT116 cells stained with propidium iodide and analysed via flow cytometry.** A minimum of 10,000 events were captured within the 'single cells gate' using a BDCantoTM II flow cytometer before further analysis using FlowJo™ v10.7.

3. Antibodies used for quantitative western blotting

Table S1: **Antibody information**.

Antibody	Dilution	Source	Clonality	Manufacturer	Product code
HDAC1	1:2000	Rabbit	Monoclonal	Abcam	ab109411
HDAC2	1:2000	Mouse	Monoclonal	Merck Millipore	05-814
HDAC3	1:2000	Rabbit	Monoclonal	Abcam	32369
H3K56Ac	1:1000	Rabbit	Polyclonal	Active Motif	39281
H2BK5Ac	1:1000	Rabbit	Polyclonal	Active Motif	39123
**Loading Controls**					
α-Tubulin	1:15000	Mouse	Monoclonal	Sigma	T5168
H3	1:1000	Mouse	Monoclonal	Merck Millipore	05-499
H2B	1:1000	Mouse	Monoclonal	Cell Signaling	2934s

IRDye®	Dilution	Source	Manufacturer	Product code
IRDye® 800CW-Mouse	1:15000	Goat anti-Mouse IgG	LICOR	926-32210
IRDye® 800CW-Rabbit	1:15000	Goat anti-Rabbit IgG	LICOR	926-32211
IRDye® 680LT-Mouse	1:15000	Goat anti-Mouse IgG	LICOR	926-68020
IRDye® 680LT-Rabbit	1:15000	Donkey anti-Rabbit IgG	LICOR	926-68023

4. Western blotting - Initial screen of JPS026, JPS027 and IAP ligand

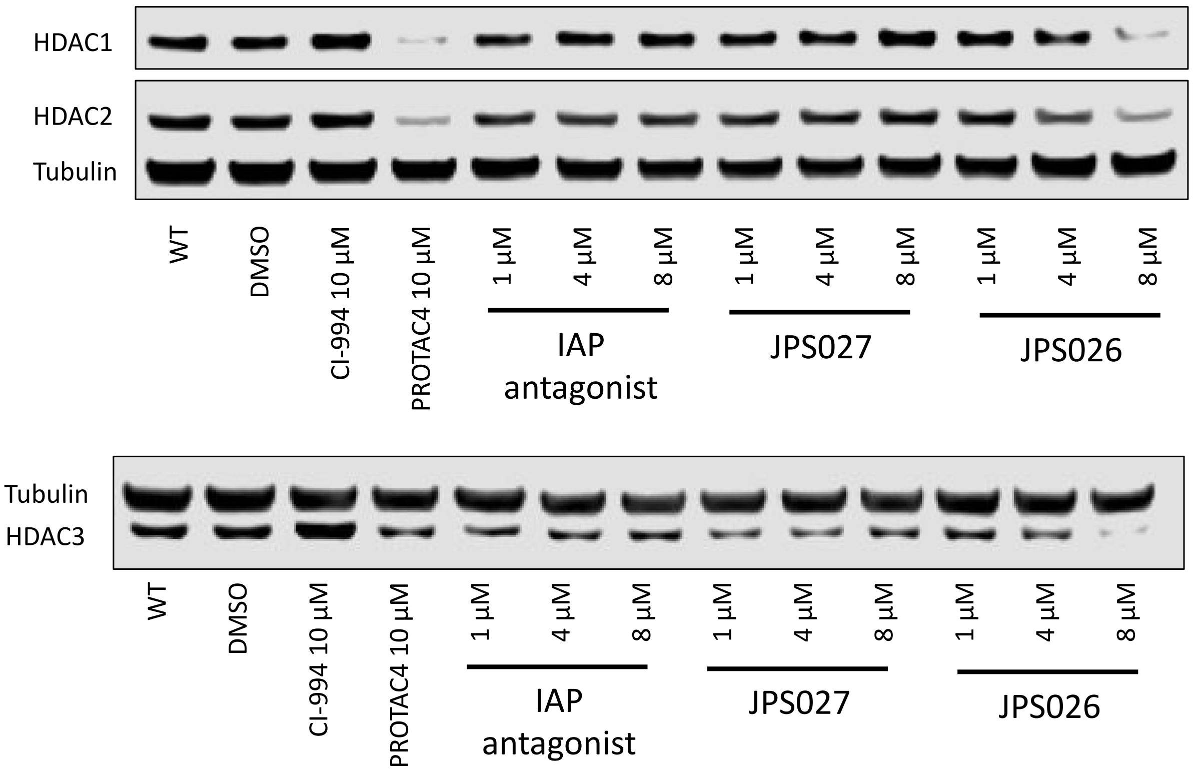

Figure S4: **Western blotting of HDAC1,2 and 3 protein levels in HCT116 cells treated with PROTACs JPS026, JPS027 and IAP ligand at 1 µM, 4 µM and 8, µM**

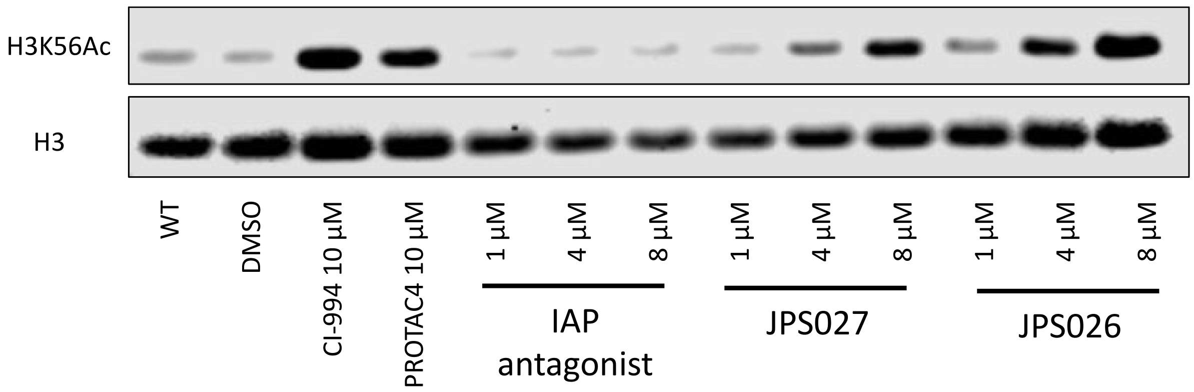

Figure S5: **Western blotting of H3/H3K56ac in HCT116 cells treated with PROTACs JPS026, JPS027 and IAP ligand 1 µM, 4 µM and 8, µM.**

Supporting Information: Chemistry

1. Materials and Methods

General Considerations

All reagents were purchased from commercially available sources and used without further purification. IAP Ligand **A 410099.1, amine** was purchased from Tocris Bioscience. Preparative column chromatography and flash column chromatography using a Biotage Isolera purification system were both performed using silica gel 60 (230−400 mesh). Biotage® Macroporous polystyrene-co-divinylbenzene (MP) carbonate resin (3.02 mmol/g loading capacity) was used for neutralizing amine TFA salts following tert-butoxycarbonyl deprotection reactions. All chemical names have been generated using ChemDraw Professional. Semipreparative HPLC was performed on a Thermo Fisher Ultimate 3000 system with Chromeleon software on a Phenomenex Luna C18 column. The mobile phases were water and acetonitrile with a flow rate of 10 mL/ min, 45 min gradient. NMR spectra were acquired using a Bruker 400 (1 H, 400 MHz; 13C 101 MHz) instrument at ambient temperature using a deuterated solvent as a reference. High-resolution mass spectra (HRMS) were recorded on a Water Aquity XEVO Q ToF machine and measured in m/z. All intermediates and final compounds were fully assigned by ^{1}H and ^{13}C NMR using 2D NMR spectra, with analysis performed using ACDLabs software (Chemsketch and Spectrus Processor).

General Methods

General Method A. To a solution of HDACi-linker acid (1.2 equiv.) in dry DMF (1 mL) at 0 °C, DIPEA (3 equiv.) and HATU (1.3 equiv.) were added. The reaction mixture was stirred for 15 minutes, after which a solution of *tert*-Butyl [(*S*)-1-[[(*S*)-2-[(2*S*,4S)-4-amino-2-[[(*R*)-1,2,3,4-tetrahydronaphthalen-1-yl]carbamoyl]pyrrolidin-1-yl]-1-cyclohexyl-2-oxoethyl]amino]-1-oxopropan-2-yl](methyl)carbamate hydrochloride (**A 410099.1, amine**, 0.038 mmol) in DMF (1 mL) was added slowly and the resultant solution stirred at room temperature for 16 hours. The reaction mixture was diluted in EtOAc (10 mL), then washed with sat. $NaHCO_3$ (2 x 5 mL) and sat. NaCl (2 x 5 mL). The organic layer was dried over $MgSO_4$, filtered, and concentrated *in vacuo* to give the corresponding crude, which was chromatographically purified to afford the desired compound.

General Method B. TFA (0.4 mL or 20 equiv.) was added to a stirring solution of Boc-protected starting material (1 equiv.) in DCM (2 mL) and the resulting reaction mixture stirred at room temperature for 4 hours. The reaction mixture was concentrated *in vacuo*, dissolved in MeOH (2 mL), agitated in MP-carbonate resin (3.02 mmol/g loading capacity) for 2-3 hours and then filtered. The filtrate was concentrated *in vacuo* and the resulting solid dissolved in MeCN:H_2O (1:1) and lyophilised to remove residual TFA impurities, affording the final compound. Prior to biological evaluation the

product was further purified by semi-preparative HPLC (5-95% MeCN in H2O, 260 nm, 45 min gradient).

2. Preparation of **JPS026** and **JPS027**

Scheme S1. Synthesis of JPS026 and JPS027.

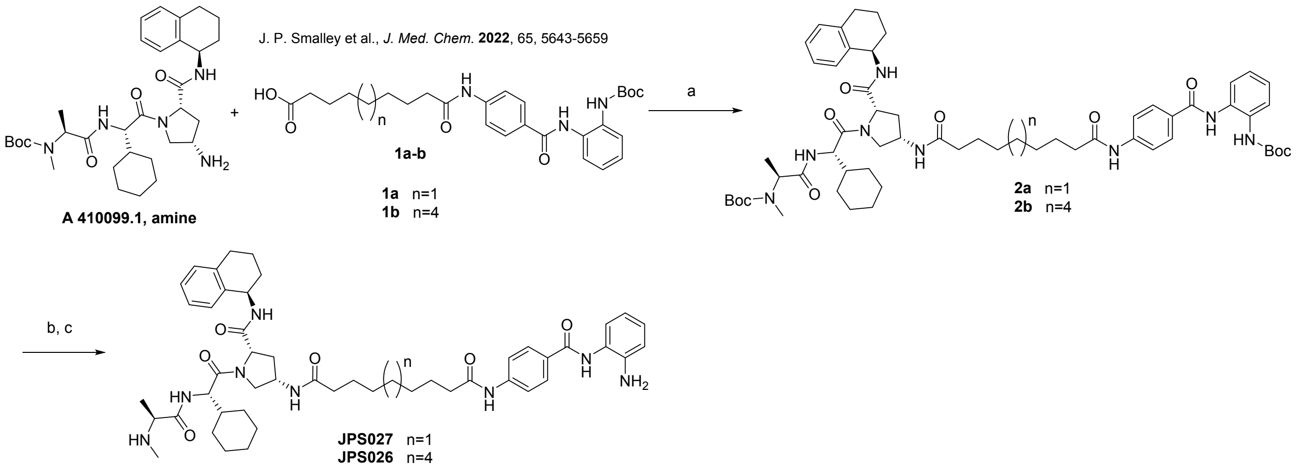

Reagents and conditions: (a) HATU, DIPEA, DMF, r.t., overnight; (b) TFA, DCM, r.t., 4 h; (c) MP-carbonate resin, MeOH, r.t., 2 h.

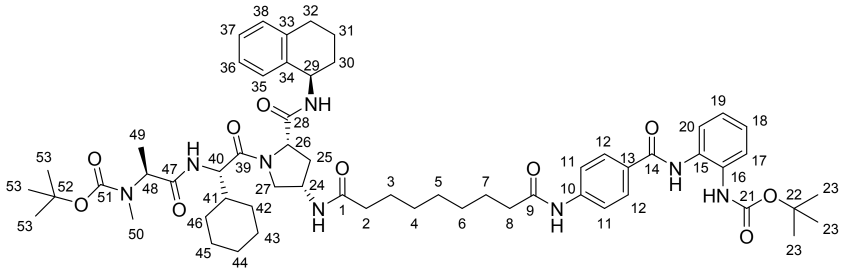

Tert-butyl ((S)-1-(((S)-2-((2S,4S)-4-(9-((4-((2-((tert-butoxycarbonyl)amino)phenyl)carbamoyl) phenyl)amino)-9-oxononanamido)-2-(((R)-1,2,3,4-tetrahydronaphthalen-1-yl)carbamoyl)pyrrolidin-1-yl)-1-cyclohexyl-2-oxoethyl)amino)-1-oxopropan-2-yl)(methyl)carbamate (2a): Following general method A, **2a** was obtained from **1a** (22.4 mg, 0.045 mmol) and **A 410099.1, amine** (24.5 mg, 0.038 mmol). The crude product was purified by column chromatography (0-10% MeOH in DCM) to afford **2a** (36.6 mg, 0.034 mmol, 91% yield) as a pale yellow solid. ^{1}H NMR (400 MHz, Methanol-*d_4*) δ_H ppm 7.93 (d, *J*=8.8 Hz, 2 H, 12-CH), 7.73 (d, *J*=8.8 Hz, 2 H, 11-CH), 7.56 - 7.63 (m, 1 H, 20-CH), 7.41 - 7.47 (m, 1 H, 17-CH), 7.35 - 7.41 (m, 1 H, 38-CH), 7.18 - 7.25 (m, 2 H, 18-CH,19-CH), 7.10 - 7.17 (m, 2 H, 36-CH,37-CH), 7.04 - 7.08 (m, 1 H, 35-CH), 5.04 (t, *J*=6.3 Hz, 1 H, 29-CH), 4.54 - 4.63 (m, 1 H, 48-CH), 4.47 - 4.54 (m, 1 H, 24-CH), 4.38 - 4.46 (m, 2 H, 26-CH,40-CH), 4.14 (dd, *J*=10.3, 6.2 Hz, 1 H, 27-CH), 3.56 (dd, *J*=10.3, 5.5 Hz, 1 H, 27-

CH), 2.84 (s, 3 H, 50-CH_3), 2.70 - 2.81 (m, 2 H, 32-CH_2), 2.45 - 2.56 (m, 1 H, 25-CH), 2.41 (t, *J*=7.4 Hz, 2 H, 8-CH_2), 2.21 (t, *J*=7.5 Hz, 2 H, 2-CH_2), 1.66 - 2.00 (m, 13 H, (7,30,31)-CH_2,(25,41,42-46)-CH), 1.60 - 1.66 (m, 2 H, 3-CH_2), 1.49 (s, 9 H, 23/53-CH_3), 1.47 (s, 9 H, 23/53-CH_3), 1.35 - 1.43 (m, 6 H, (4-6)-CH_2), 1.31 (d, *J*=7.1 Hz, 3 H, 49-CH_3), 1.16 - 1.29 (m, 3 H, (43-45)-CH), 1.00 - 1.15 (m, 2 H, 42-CH,46-CH). ^{13}C NMR (101 MHz, Methanol-d_4) δ_C ppm 175.8 (C1), 175.0 (C9), 174.4 (C47), 173.5 (C28), 172.8 (C39), 167.9 (C14), 157.7 (C51), 156.4 (C21), 143.9 (C10), 138.7 (C34), 137.7 (C33), 133.2 (C16), 131.8 (C15), 130.3 (C13), 130.1 (C35), 129.9 (C38), 129.7 (C12), 128.3 (C36), 127.5 (C18/19), 127.3 (C37), 127.25 (C20), 126.4 (C18/19), 125.7 (C17), 120.4 (C11), 81.9 (C23,C53), 60.9 (C26), 57.3 (C40), 55.4 (C48), 54.6 (C27), 50.0 (C24), 49.3 (C29), 41.7 (C41), 38.2 (C8), 37.4 (C2), 35.8 (C25), 31.4 (C30), 31.2 (C50), 30.7 (C42/46), 30.4 (C32), 30.3 (C4/5/6), 30.25 (C4/5/6) 30.2 (C4/5/6), 30.1 (C42/46), 28.9 (C23/53), 28.8 (C23/53), 27.4 (C43/44/45), 27.4 (C43/44/45), 27.2 (C43/44/45), 26.9 (C3), 26.8 (C7), 21.8 (C31), 14.6 (C49). HRMS (ESI) m/z: $[M+H]^+$ calculated for $C_{59}H_{83}N_8O_{10}$: 1063.6232, found 1063.6194.

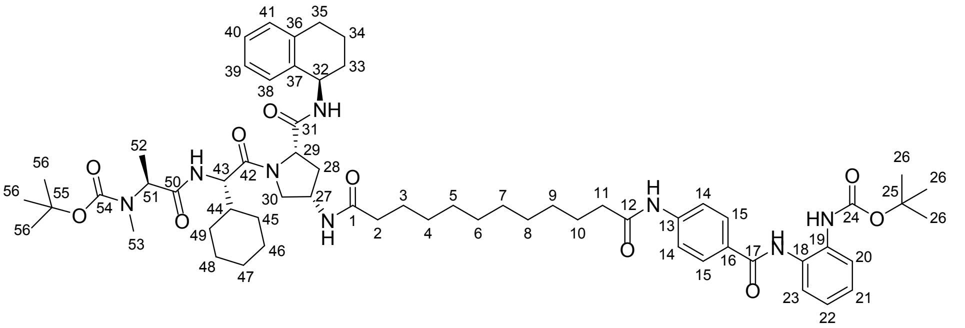

Tert-butyl ((S)-1-(((S)-2-((2S,4S)-4-(12-((4-((2-((tert-butoxycarbonyl)amino)phenyl)carbamoyl) phenyl)amino)-12-oxododecanamido)-2-(((R)-1,2,3,4-tetrahydronaphthalen-1-yl)carbamoyl)pyrrolidin-1-yl)-1-cyclohexyl-2-oxoethyl)amino)-1-oxopropan-2-yl)(methyl)carbamate (2b): Following general method A, **2b** was obtained from **1b** (24.3 mg, 0.045 mmol) and **A 410099.1, amine** (24.5 mg, 0.038 mmol). The crude product was purified by column chromatography (0-10% MeOH in DCM) to afford **2b** (27.3 mg, 0.025 mmol, 65% yield) as a pale yellow solid. ^{1}H NMR (400 MHz, Methanol-d_4) δ_H ppm 7.93 (d, *J*=8.8 Hz, 2 H, 15-CH), 7.73 (d, *J*=8.8 Hz, 2 H, 14-CH), 7.56 - 7.63 (m, 1 H, 23-CH), 7.41 - 7.47 (m, 1 H, 20-CH), 7.35 - 7.41 (m, 1 H, 41-CH), 7.17 - 7.26 (m, 2 H, 21,22-CH), 7.11 - 7.15 (m, 2 H, 39,40-CH), 7.05 - 7.09 (m, 1 H, 38-CH), 5.02 - 5.08 (m, 1 H, 32-CH), 4.54 - 4.63 (m, 1 H, 51-CH), 4.48 - 4.54 (m, 1 H, 27-CH), 4.38 - 4.46 (m, 2 H, 29,43-CH), 4.13 (dd, *J*=10.3, 6.2 Hz, 1 H, 30-CH), 3.57 (dd, *J*=10.3, 5.4 Hz, 1 H, 30-CH), 2.84 (s, 3 H, 53-CH_3), 2.71 - 2.81 (m, 2 H, 35-CH_2), 2.46 - 2.55 (m, 1 H, 28-CH), 2.40 (t, *J*=7.5 Hz, 2 H, 11-CH_2), 2.19 (t, *J*=7.5 Hz, 2 H, 2-CH_2), 1.68 - 1.98 (m, 13 H, 10,33,34-CH_2,28,44,(45-49)-CH), 1.58 - 1.64 (m, 2 H, 3-CH_2), 1.50 (s, 9 H, 26/56-CH_3), 1.47 (s, 9 H, 26/56-CH_3), 1.32 - 1.41 (m, 12 H, (4-9)-CH_2), 1.01-1.29 (m, 8 H, (46-48)-CH,52-CH_3). ^{13}C NMR (101 MHz, Methanol-d_4) δ_C ppm 175.9 (C1), 175.1 (C12),

174.4 (C50), 173.5 (C31), 172.8 (C42), 167.9 (C17), 157.6 (C54), 156.4 (C24), 143.9 (C13), 138.7 (C37), 137.7 (C36), 133.2 (C19), 131.9 (C18), 130.3 (C16), 130.1 (C38), 129.9 (C41), 129.7 (C15), 128.3 (C39), 127.5 (C21/22), 127.3 (C40), 127.25 (C23), 126.4 (C21/22), 125.7 (C20), 120.4 (C14), 81.9 (C25,55), 60.9 (C29), 57.3 (C43), 55.3 (C51), 54.6 (C30), 50.1 (C27), 49.2 (C32), 41.7 (C44), 38.2 (C11), 37.5 (C2), 35.8 (C28), 31.4 (C33), 31.2 (C53), 30.75 (C45/49), 30.7 (alkyl CH_2), 30.65 (alkyl CH_2), 30.6 (alkyl CH_2), 30.55 (alkyl CH_2), 30.5 (alkyl CH_2), 30.45 (alkyl CH_2), 30.4 (C35), 30.2 (C45/49), 28.9 (C26/56), 28.8 (C26/56), 27.45 (C46/47/48), 27.4 (C46/47/48), 27.2 (C46/47/48), 27.0 (C3), 26.9 (C10), 21.8 (C34), 14.6 (C52). HRMS (ESI) m/z: $[M+H]^+$ calculated for $C_{62}H_{89}N_8O_{10}$: 1105.6702, found 1105.6704.

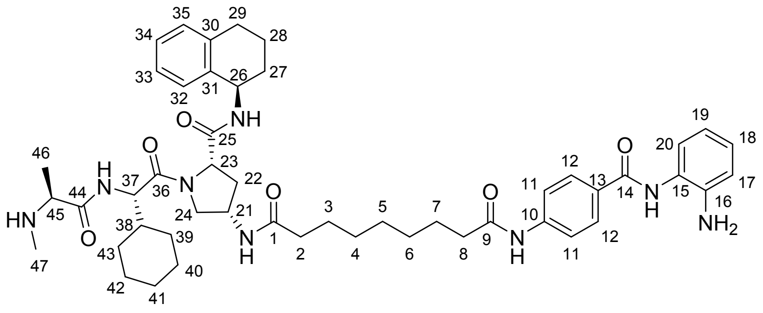

N1-(4-((2-aminophenyl)carbamoyl)phenyl)-N9-((3S,5S)-1-((S)-2-cyclohexyl-2-((S)-2-(methylamino) propanamido)acetyl)-5-(((R)-1,2,3,4-tetrahydronaphthalen-1-yl)carbamoyl)pyrrolidin-3-yl)nonanediamide (JPS027): Following general method B, dual Boc deprotection of **2a** (36.6 mg, 0.034 mmol) was performed to afford **JPS027** (25.8 mg, 0.030 mmol, 87% yield) as a white solid. ^{1}H NMR (400 MHz, Methanol-d_4) δ_H ppm 7.95 (d, J=8.7 Hz, 2 H, 12-CH), 7.73 (d, J=8.7 Hz, 2 H, 11-CH), 7.35 - 7.42 (m, 1 H, 35-CH), 7.18 (dd, J=7.8, 1.2 Hz, 1 H, 20-CH), 7.10 - 7.16 (m, 2 H, 33-CH,34-CH), 7.05 - 7.10 (m, 2 H, 18-CH,32-CH), 6.90 (dd, J=7.8, 1.2 Hz, 1 H, 17-CH), 6.77 (app. td, J=7.8, 1.2 Hz, 1 H, 19-CH), 5.04 (br t, J=5.9 Hz, 1 H, 26-CH), 4.41 - 4.53 (m, 3 H, (21,23,37)-CH), 4.18 (dd, J=10.3, 6.3 Hz, 1 H, 24-CH), 3.57 (dd, J=10.3, 5.6 Hz, 1 H, 24-CH), 3.18 (q, J=6.8 Hz, 1 H, 45-CH), 2.73 - 2.84 (m, 2 H, 29-CH_2), 2.51 (ddd, J=13.1, 8.5, 6.6 Hz, 1 H, 22-CH), 2.41 (t, J=7.4 Hz, 2 H, 8-CH_2), 2.31 (s, 3 H, 47-CH_3), 2.21 (t, J=7.2 Hz, 2 H, 2-CH_2), 1.68 - 2.00 (m, 13 H, (7,27,28)-CH_2,(22,38,39-43)-CH), 1.61 - 1.67 (m, 2 H, 3-CH_2), 1.36 - 1.45 (m, 6 H, (4-6)-CH_2), 1.27 - 1.31 (m, 3 H, (40-42)-CH), 1.22 (d, J=6.8 Hz, 3 H, 46-CH_3), 1.03 - 1.14 (m, 2 H, 39-CH,43-CH). ^{13}C NMR (101 MHz, Methanol-d_4) δ_C ppm 177.4 (C44), 175.9 (C1), 175.0 (C9), 173.6 (C25), 172.8 (C36), 168.4 (C14), 143.9 (C16), 143.7 (C10), 138.7 (C31), 137.7 (C30), 130.5 (C13), 130.1 (C32), 130.0 (C35), 129.9 (C12), 128.6 (C18), 128.3 (C33), 127.8 (C20), 127.3 (C34), 125.6 (C15), 120.4 (C11), 119.8 (C19), 118.9 (C17), 60.9 (C23), 60.3 (C45), 57.1 (C37), 54.6 (C24), 50.0 (C21), 49.3 (C26), 41.6 (C38), 38.2 (C8), 37.4 (C2), 35.8 (C22), 34.7 (C47), 31.4 (C27), 30.9 (C39/43), 30.7 (C29), 30.4 (C4/5/6), 30.2 (C4/5/6), 30.2 (C4/5/6), 30.1 (C39/43), 27.4 (C40/41/42), 27.4 (C40/41/42), 27.2

(C40/41/42), 26.9 (C3), 26.9 (C7), 21.7 (C28), 19.5 (C46). HRMS (ESI) m/z: $[M+H]^+$ calculated for $C_{49}H_{67}N_8O_6$: 863.5184, found 863.5160.

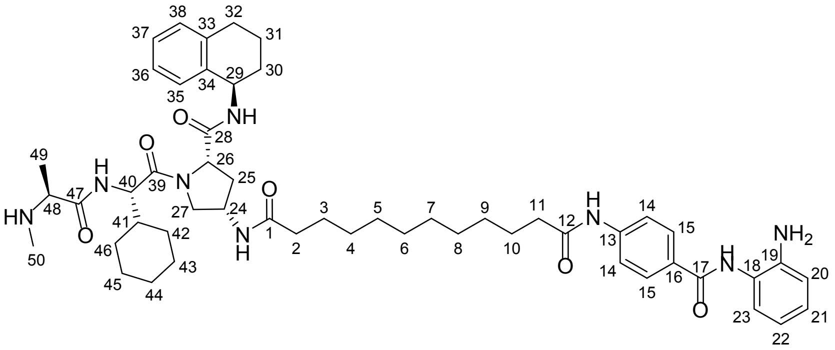

N1-(4-((2-aminophenyl)carbamoyl)phenyl)-N12-((3S,5S)-1-((S)-2-cyclohexyl-2-((S)-2-(methylamino) propanamido)acetyl)-5-(((R)-1,2,3,4-tetrahydronaphthalen-1-yl)carbamoyl)pyrrolidin-3-yl) dodecanediamide (JPS026): Following general method B, dual Boc deprotection of **2b** (27.3 mg, 0.025 mmol) was performed to afford **JPS026** (22.3 mg, 0.024 mmol, 99% yield) as a white solid. ^1H NMR (400 MHz, Methanol-d_4) δ_H ppm 7.96 (d, J=8.7 Hz, 2 H, 15-CH), 7.73 (d, J=8.7 Hz, 2 H, 14-CH), 7.34 - 7.42 (m, 1 H, 38-CH), 7.18 (dd, J=7.8, 1.2 Hz, 1 H, 23-CH), 7.10 - 7.16 (m, 2 H, 36,37-CH), 7.05 - 7.10 (m, 2 H, 21,35-CH), 6.90 (dd, J=7.8, 1.2 Hz, 1 H, 20-CH), 6.77 (app. td, J=7.8, 1.2 Hz, 1 H, 22-CH), 5.03 - 5.07 (m, 1 H, 29-CH), 4.40 - 4.54 (m, 3 H, 24,26,40-CH), 4.17 (dd, J=10.3, 6.2 Hz, 1 H, 27-CH), 3.57 (dd, J=10.3, 5.4 Hz, 1 H, 27-CH), 3.24 (q, J=6.8 Hz, 1 H, 48-CH), 2.74 - 2.86 (m, 2 H, 32-CH_2), 2.50 (ddd, J=13.2, 8.7, 6.7 Hz, 1 H, 25-CH), 2.40 (t, J=7.4 Hz, 2 H, 11-CH_2), 2.35 (s, 3 H, 50-CH_3), 2.20 (t, J=7.5 Hz, 2 H, 2-CH_2), 1.66 – 2.03 (m, 13 H, 10,30,31-CH_2,25,41,(42-46)-CH), 1.59 - 1.65 (m, 2 H, 3-CH_2), 1.32 - 1.42 (m, 12 H, (4-9)-CH_2), 1.27-1.32 (m, 3 H, (43-45)-CH), 1.25 (d, J=6.8 Hz, 3 H, 49-CH_3), 1.04 - 1.14 (m, 2 H, 42,46-CH). ^{13}C NMR (101 MHz, Methanol-d_4) δ_C ppm 177.4 (C47), 175.9 (C1), 175.1 (C12), 173.6 (C28), 172.8 (C39), 168.4 (C17), 144.0 (C19), 143.7 (C13), 138.7 (C34), 137.7 (C33), 130.5 (C16), 130.1 (C35), 130.0 (C38), 129.9 (C15), 128.6 (C21), 128.3 (C36), 127.8 (C23), 127.3 (C37), 125.6 (C18), 120.4 (C14), 119.8 (C22), 118.9 (C20), 60.9 (C26), 60.3 (C48), 57.1 (C40), 54.6 (C27), 50.1 (C24), 49.3 (C29), 41.6 (C41), 38.2 (C11), 37.5 (C2), 35.8 (C25), 34.7 (C50), 31.4 (C30), 30.75 (C42/46), 30.7 (alkyl CH_2), 30.6 (alkyl CH_2), 30.55 (alkyl CH_2), 30.5 (alkyl CH_2), 30.45 (alkyl CH_2), 30.4 (alkyl CH_2), 30.35 (C32), 30.1 (C42/46), 27.45 (C43/44/45), 27.4 (C43/44/45), 27.2 (C43/44/45), 27.0 (C3), 26.9 (C10), 21.7 (C31), 19.5 (C49). HRMS (ESI) m/z: $[M+H]^+$ calculated for $C_{52}H_{73}N_8O_6$: 905.5653, found 905.5648.

3. Preparation of **IAP Ligand**

Scheme S2. Synthesis of IAP Ligand.

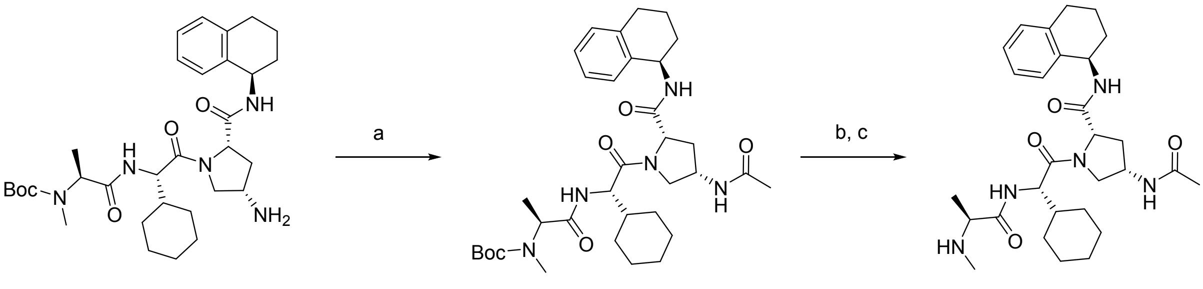

A 410099.1, amine | **3** | **IAP Ligand**

Reagents and conditions: (a) Acetic anhydride, NEt_3, DMF, 0 °C - r.t., 2 h; (b) TFA, DCM, r.t., 4 h; (c) MP-carbonate resin, MeOH, r.t., 2 h.

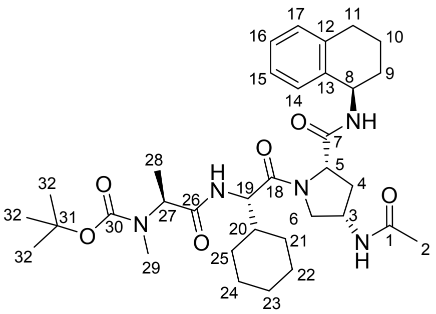

Tert-butyl ((S)-1-(((S)-2-((2S,4S)-4-acetamido-2-(((R)-1,2,3,4-tetrahydronaphthalen-1-yl)carbamoyl) pyrrolidin-1-yl)-1-cyclohexyl-2-oxoethyl)amino)-1-oxopropan-2-yl)(methyl)carbamate (3): To a solution of **A 410099.1 amine** (24.5 mg, 0.038 mmol) in DMF (1 mL) at 0ºC, NEt_3 (16 µL, 0.113 mmol) was added and the reaction mixture stirred at room temperature for 10 minutes. Acetic anhydride (5.3 µL, 0.056 mmol) was then added and the reaction mixture stirred at room temperature for 2 hours. The reaction mixture was concentrated *in vacuo* and then purified by column chromatography (0-10% MeOH in DCM) to afford **5** (22.3 mg, 0.036 mmol, 95% yield) as a white solid. ^{1}H NMR (400 MHz, Chloroform-*d*) δ_H ppm 7.98 (d, *J*=6.4 Hz, 1 H, 1-NH), 7.76 (d, *J*=5.9 Hz, 1 H, 7-NH), 7.12 - 7.18 (m, 2 H, 14-CH,16-CH), 7.05 - 7.11 (m, 2 H, 15-CH,17-CH), 6.62 (br s, 1 H, 26-NH), 5.08 - 5.15 (m, 1 H, 8-CH), 4.70 - 4.75 (m, 1 H, 5-CH), 4.62 - 4.70 (m, 1 H, 27-CH), 4.59 (q, *J*=6.4 Hz, 1 H, 3-CH), 4.33 (t, *J*=7.8 Hz, 1 H, 19-CH), 4.01 (dd, *J*=10.9, 5.1 Hz, 1 H, 6-CH), 3.67 (d, *J*=10.9 Hz, 1 H, 6-CH), 2.70 - 2.86 (m, 5 H, 11-CH_2,29-CH_3), 2.36 - 2.43 (m, 1 H, 4-CH), 2.14 - 2.22 (m, 1 H, 4-CH), 1.99 - 2.06 (m, 4 H, 9-CH,2-CH_3), 1.80 - 1.90 (m, 3 H, 9-CH,10-CH_2), 1.51 - 1.71 (m, 6 H, (20-25)-CH), 1.46 (s, 9 H, 32-CH_3), 1.28 (d, *J*=7.2 Hz, 3 H, 28-CH_3), 1.02 - 1.13 (m, 3 H, (22-24)-CH), 0.84 - 0.97 (m, 2 H, 21-CH,25-CH). ^{13}C NMR (101 MHz, Chloroform-*d*) δ_C ppm 172.9 (C18), 171.4 (C26), 171.1 (C7), 170.0 (C1), 156.4 (C30), 137.2 (C12), 136.0 (C13), 129.2 (C14/16), 128.2

(C15/17), 127.3 (C15/17), 126.0 (C14/16), 80.6 (C31), 60.1 (C5), 55.6 (C6), 55.2 (C19), 53.1 (C27), 49.5 (C3), 47.9 (C8), 40.5 (C20), 31.1 (C4), 29.8 (C29), 29.7 (C9), 29.2 (C21/25), 29.1 (C11), 28.4 (C21/25), 28.3 (C32), 25.9 (C22/23/24), 25.85 (C22/23/24), 25.8 (C22/23/24), 23.3 (C2), 19.9 (C10), 13.2 (C28). HRMS (ESI) m/z: $[M+H]^+$ calculated for $C_{34}H_{52}N_5O_6$: 626.3918, found 626.3917.

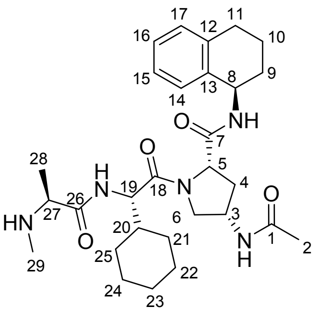

(2S,4S)-4-acetamido-1-((S)-2-cyclohexyl-2-((S)-2-(methylamino)propanamido)acetyl)-N-((R)-1,2,3,4-tetrahydronaphthalen-1-yl)pyrrolidine-2-carboxamide (IAP Ligand): Following general method B, Boc deprotection of **3** (22.3 mg, 0.036 mmol) was performed to afford **IAP Ligand** (18.4 mg, 0.035 mmol, 98% yield) as a white solid. ^{1}H NMR (400 MHz, Methanol-*d₄)* δ_H ppm 7.37 - 7.43 (m, 1 H, 17-CH), 7.11 - 7.17 (m, 2 H, 15-CH,16-CH), 7.06 - 7.10 (m, 1 H, 14-CH), 5.05 (br t, *J*=5.9 Hz, 1 H, 8-CH), 4.39 - 4.51 (m, 3 H, (3,5,19)-CH), 4.22 (dd, *J*=10.2, 6.6 Hz, 1 H, 6-CH), 3.53 (dd, *J*=10.2, 6.6 Hz, 1 H, 6-CH), 3.15 (q, *J*=6.9 Hz, 1 H, 27-CH), 2.72 - 2.85 (m, 2 H, 11-CH_2), 2.52 (ddd, *J*=12.9, 8.5, 6.7 Hz, 1 H, 4-CH), 2.30 (s, 3 H, 29-CH_3), 1.97 - 2.03 (m, 1 H, 9-CH), 1.96 (s, 3 H, 2-CH_3), 1.67 - 1.93 (m, 10 H,10-CH_2,(4,9,20,(21-25)-CH), 1.26 - 1.32 (m, 3 H, (22,23,24)-CH), 1.22 (d, *J*=6.9 Hz, 3 H, 28-CH_3), 1.05 - 1.16 (m, 2 H, 21-CH,25-CH). ^{13}C NMR (101 MHz, Methanol-d_4) δ_C ppm 177.3 (C26), 173.4 (C7), 173.0 (C1), 172.7 (C18), 138.7 (C13), 137.7 (C12), 130.1 (C14), 130.0 (C17), 128.3 (C15), 127.3 (C16), 60.8 (C5), 60.3 (C27), 57.1 (C19), 54.1 (C6), 50.0 (C3), 49.3 (C8), 41.5 (C20), 35.8 (C4), 34.6 (C29), 31.4 (C9), 30.7 (C11), 30.4 (C21/25), 30.0 (C21/25), 27.4 (C22/23/24), 27.3 (C22/23/24), 27.2 (C22/23/24), 22.8 (C2), 21.7 (C10), 19.4 (C28).HRMS (ESI) m/z: $[M+H]^+$ calculated for $C_{29}H_{44}N_5O_4$: 526.3393, found 526.3397.

Appendix: ^{1}H NMR and ^{13}C NMR of Novel Compounds

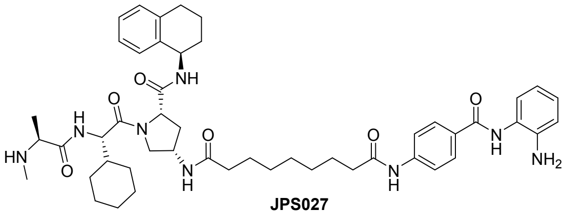

JPS027

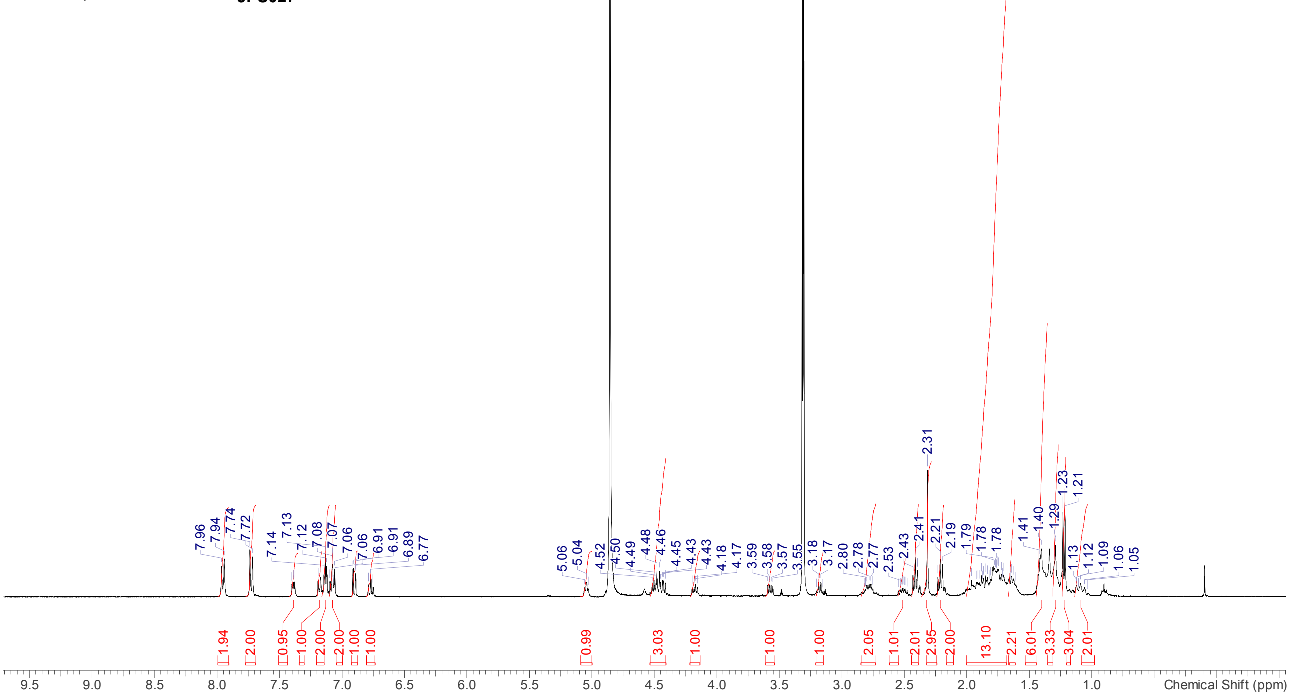

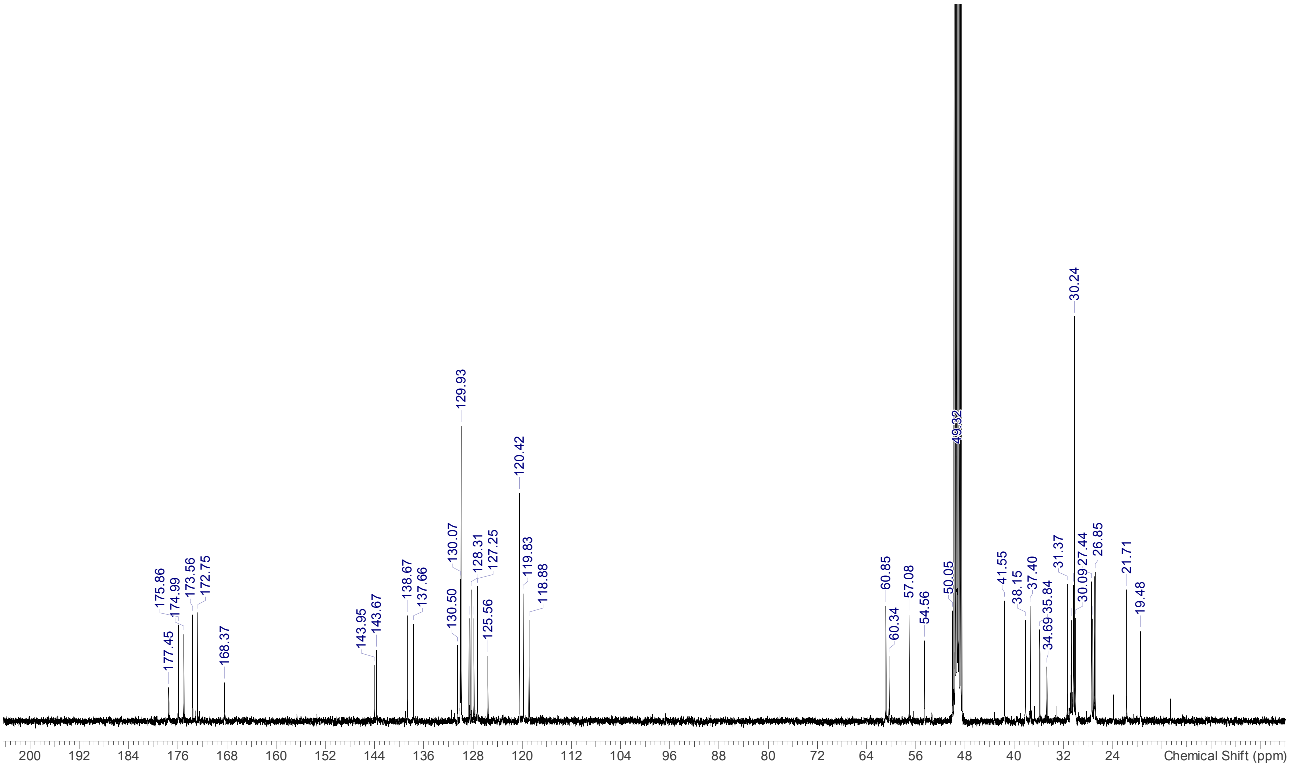

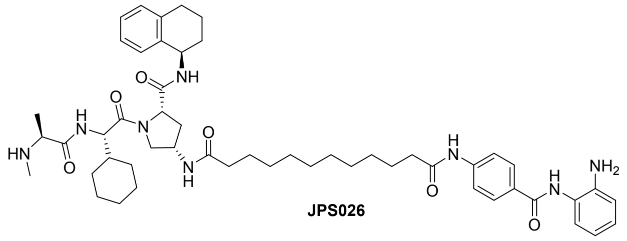

JPS026

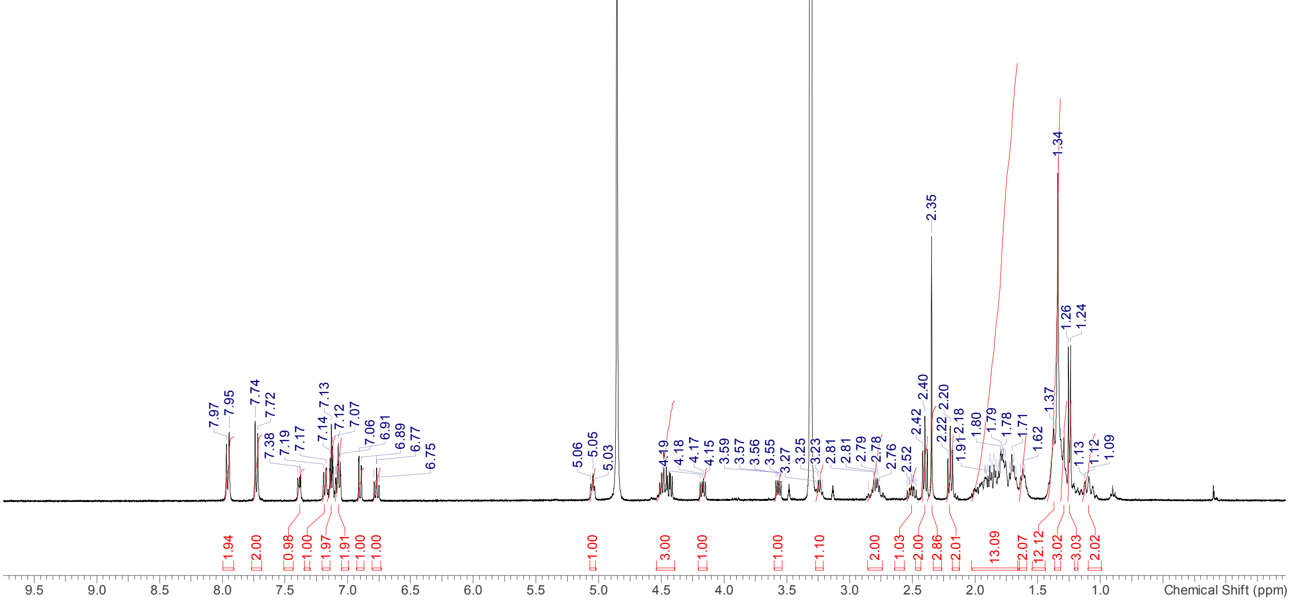

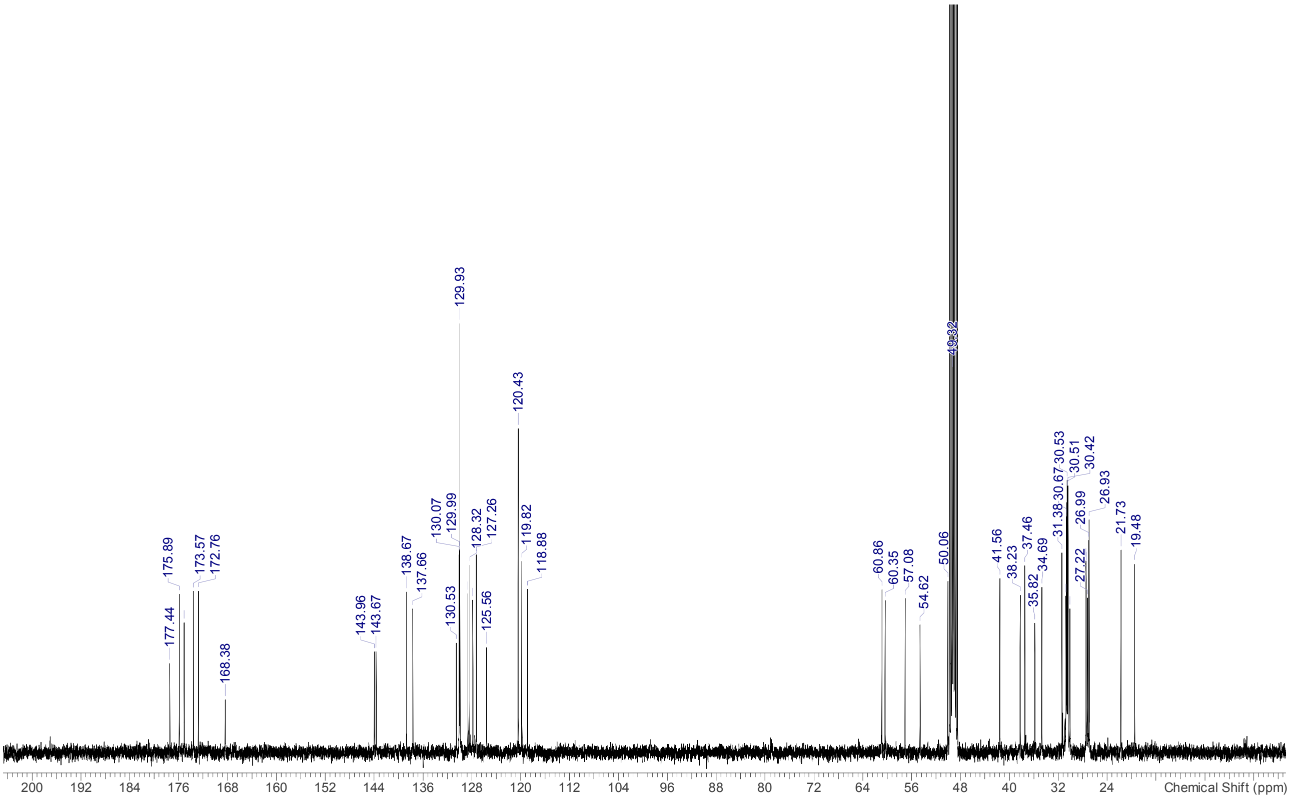

Supplement: Supplementary file 1 — bi2c00288_si_001.pdf [file bi2c00288_si_001.pdf]
